# Cost-effectiveness of cardiovascular imaging for stable coronary heart disease

**DOI:** 10.1136/heartjnl-2020-316990

**Published:** 2020-08-14

**Authors:** Simon Walker, Edward Cox, Ben Rothwell, Colin Berry, Gerry P McCann, Chiara Bucciarelli-Ducci, Erica Dall’Armellina, Abhiram Prasad, James Robert John Foley, Kenneth Mangion, Petra Bijsterveld, Colin Everett, Deborah Stocken, Sven Plein, John P Greenwood, Mark Sculpher

**Affiliations:** 1 Centre for Health Economics, University of York, York, UK; 2 RTI Health Solutions Manchester, Manchester, UK; 3 Institute of Cardiovascular and Medical Sciences, College of Medical, Veterinary and Life Sciences, University of Glasgow, Glasgow, UK; 4 Department of Cardiovascular Sciences, University of Leicester, Leicester, UK; 5 NIHR Leicester Cardiovascular Biomedical Research Centre, Glenfield Hospital, Leicester, UK; 6 Bristol Heart Institute, NIHR Bristol Cardiovascular Biomedical Research Centre and Clinical Research and Imaging Centre (CRIC), University of Bristol and University Hospitals Bristol NHD Trust, Bristol, UK; 7 Department of Biomedical Imaging Science, Leeds Institute of Cardiovascular & Metabolic Medicine, University of Leeds, Leeds, UK; 8 Oxford Centre of Cardiovascular Magnetic Resonance, Oxford University, Oxford, UK; 9 Department of Cardiovascular Diseases, Mayo Clinic, Rochester, Minnesota, USA; 10 Cardiovascular Sciences Research Centre, St George's, University of London, London, UK; 11 CTRU – Clinical Trials Research Unit, Leeds Institute for Clinical Trials Research, University of Leeds, Leeds, UK

**Keywords:** health care economics, cardiac magnetic resonance (CMR) imaging, cardiac imaging and diagnostics

## Abstract

**Objective:**

To assess the cost-effectiveness of management strategies for patients presenting with chest pain and suspected coronary heart disease (CHD): (1) cardiovascular magnetic resonance (CMR); (2) myocardial perfusion scintigraphy (MPS); and (3) UK National Institute for Health and Care Excellence (NICE) guideline-guided care.

**Methods:**

Using UK data for 1202 patients from the Clinical Evaluation of Magnetic Resonance Imaging in Coronary Heart Disease 2 trial, we conducted an economic evaluation to assess the cost-effectiveness of CMR, MPS and NICE guidelines. Health outcomes were expressed as quality-adjusted life-years (QALYs), and costs reflected UK pound sterling in 2016–2017. Cost-effectiveness results were presented as incremental cost-effectiveness ratios and incremental net health benefits overall and for low, medium and high pretest likelihood of CHD subgroups.

**Results:**

CMR had the highest estimated QALY gain overall (2.21 (95% credible interval 2.15, 2.26) compared with 2.07 (1.92, 2.20) for NICE and 2.11 (2.01, 2.22) for MPS) and incurred comparable costs (overall £1625 (£1431, £1824) compared with £1753 (£1473, £2032) for NICE and £1768 (£1572, £1989) for MPS). Overall, CMR was the cost-effective strategy, being the dominant strategy (more effective, less costly) with incremental net health benefits per patient of 0.146 QALYs (−0.18, 0.406) compared with NICE guidelines at a cost-effectiveness threshold of £15 000 per QALY (93% probability of cost-effectiveness). Results were similar in the pretest likelihood subgroups.

**Conclusions:**

CMR-guided care is cost-effective overall and across all pretest likelihood subgroups, compared with MPS and NICE guidelines.

## Introduction

Coronary heart disease (CHD) is a leading cause of morbidity and mortality worldwide. In patients presenting with chest pain, a range of invasive and non-invasive tests are available for the diagnosis of CHD and are used to identify patients suitable for revascularisation. While myocardial perfusion scintigraphy (MPS) is the most commonly used non-invasive functional imaging test worldwide, cardiovascular magnetic resonance (CMR) has been shown to have higher diagnostic accuracy and prognostic value.^1 2^ Despite the widespread availability of non-invasive imaging, invasive coronary angiography (ICA) remains frequently used in the diagnostic pathway for stable angina, despite its higher cost and associated risks. US and UK studies have shown that after ICA, a large proportion of patients with stable chest pain are found not to have significant obstructive disease,^3 4^ suggesting that ICA may be unnecessary and potentially avoidable in many.

The optimal initial investigation strategy in patients with stable chest pain remains keenly debated, and the cost-effectiveness of different strategies is also unclear. These are likely to depend on the pretest likelihood of having CHD.^5–7^ Updated UK clinical guidelines for the diagnosis of stable chest pain recommended the use of CT-guided care, but used non-standard approaches for cost-effectiveness evaluation.^8 9^ Furthermore, the cost-effectiveness of CT-guided management remains uncertain.^10^

The Clinical Evaluation of Magnetic Resonance Imaging in Coronary Heart Disease 2 (CE-MARC 2) trial compared three diagnostic strategies in patients presenting with stable chest pain and suspected CHD. The aim of this protocol-defined, prespecified analysis was to use data from CE-MARC 2 trial to assess the cost-effectiveness, from a UK National Health Service (NHS) perspective, of CMR, MPS and a risk-stratified approach based on the UK National Institute for Health and Care Excellence (NICE) guidelines (CG95, 2010) at the time of the trial.

## Methods

### Patient and public involvement

Patients and public advisors (1) were involved in the trial design and funding application, (2) were members of the trial steering committee and (3) oversaw the drafting of all patient-facing materials.

### Study design

**Figure 1:**
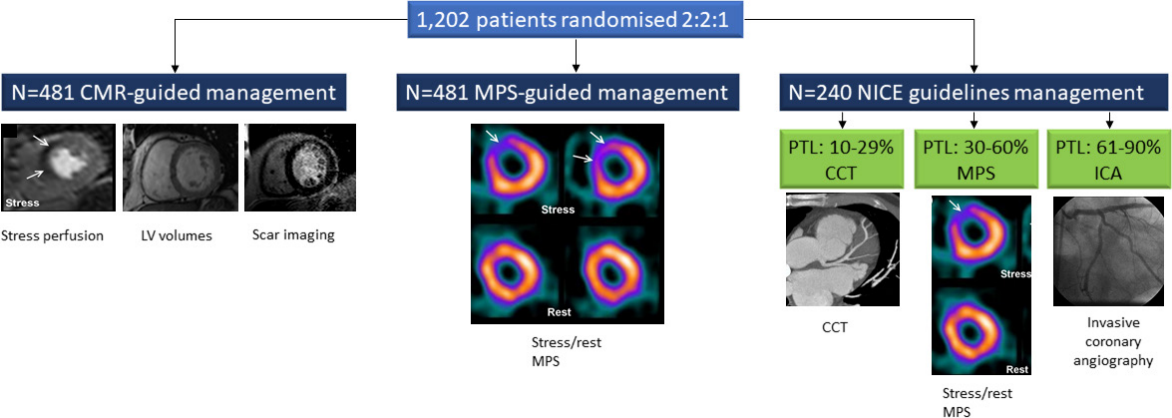
CE-MARC 2 study flow diagram illustrating randomisation and investigative strategies. CE-MARC 2, Clinical Evaluation of Magnetic Resonance Imaging in Coronary Heart Disease 2; CMR, cardiovascular magnetic resonance; LV, left ventricular; ICA, invasive coronary angiography; MPS, myocardial perfusion scintigraphy; NICE, National Institute for Health and Care Excellence; PTL, pretest likelihood; CCT, cardiac computed tomography.

The economic evaluation compared alternative diagnostic strategies in terms of their health effects based on quality-adjusted life-years (QALYs) and their costs from a UK NHS perspective over 36 months based on trial follow-up. Key data were taken from CE-MARC 2, the full details of which have been published.^11 12^ Briefly, CE-MARC 2 was a UK multicentre, three-arm, randomised controlled trial of patients with suspected angina pectoris, 30 years or older with a 10%–90% pretest likelihood of CHD and considered suitable for coronary revascularisation.^11 12^ The trial compared three management strategies: (1) CMR-guided care, (2) MPS-guided care and (3) UK NICE guidelines (CG95, 2010).^13^ Patients randomised to NICE guidelines care were stratified according to their pretest likelihood: those with a 10%–29% (low) pretest likelihood were scheduled for cardiac CT (CCT); those with a 30%–60% (medium) pretest likelihood were scheduled for MPS; and those with a 61%–90% (high) pretest likelihood were sent directly to ICA. A positive scan (CMR, MPS, CCT) resulted in protocol-defined ICA and fractional flow reserve measurement. In this study, 1202 patients were randomised 2:2:1 to CMR, MPS and NICE guidelines guided care, respectively. Figure 1 illustrates the study flow diagram and investigative strategies. The study was conducted in accordance with the protocol which was approved by the UK National Research Ethics Service (12/YH/0404) and institutional review boards of participating centres. Informed written consent was obtained from all participants.

### Resource use and costs

Data on diagnostic and revascularisation-related resource use were collected using individual case record forms. Data relating to outpatient visits, inpatient hospitalisation and cardiovascular medication were collected at annual follow-up.

Costs reflected unit costs in pound sterling at 2016–2017 prices,^14–16^ and were grouped into diagnostic costs, revascularisation costs (percutaneous coronary intervention (PCI) or coronary artery bypass grafting (CABG)), inpatient costs (cardiovascular-related hospitalisations), outpatient costs, drug costs and total costs. To reduce the risk of spurious cost differences resulting from random imbalance in the revascularisation procedures administered in each trial arm (CABG or PCI), a weighted average revascularisation cost was applied.

Costs for the diagnostic procedures were based on NHS reference costs.^14^ Scenario analyses considered revised MPS unit costs (which do not account for costing the stress and rest procedures separately) and tariff prices (hospital reimbursement rates).^17^ Unit costs for the diagnostic tests and revascularisations are shown in table 1. Other unit costs are shown in online supplementary tables S1 and S2.

10.1136/heartjnl-2020-316990.supp1 Supplementary data

**Table 1:** Base-case primary diagnostic and revascularisation unit costs^14^

	Unit cost
**Diagnostic tests**	
Cardiac CT	£264.16
Cardiovascular magnetic resonance	£393.71
Myocardial perfusion scintigraphy	£588.94
Invasive coronary angiography	£1068.47
**Revascularisation procedures**	
Percutaneous coronary intervention	£3124.81
Coronary artery bypass grafting	£11 046.22
Average revascularisation cost	£5285.19

### Outcomes

Health outcomes were expressed as QALY, a generic measure of health capturing survival and health-related quality of life. These were estimated based on patients’ responses to the EQ-5D-3L questionnaire at baseline and at 6, 12, 24 and 36 months postrandomisation.^18^ The EQ-5D-3L asks patients to rate their health (no problems, moderate problems or severe problems) in the following categories: mobility, self-care, usual activity, pain/discomfort and anxiety/depression. When combined, these ratings define health states that have been assigned values based on the preferences of a representative sample of the UK population.^19^ The EQ-5D scores and survival data were combined to estimate QALYs using the area under the curve method with linear interpolation between time points.^20^ As an alternative scenario, the EQ-5D-5L questionnaire and its associated values were used.^21 22^

### Analysis

The analysis assessed both an overall comparison of the trial arms and a stratified analysis for each pretest likelihood stratum (low, medium, high), since previous research indicates the expected prognostic value of a diagnostic strategy is dependent on pretest likelihood.^5–7^ Costs and QALYs were discounted at 3.5% per annum.^9^ Where data items were missing, multiple imputation was used to estimate costs by category, and EQ-5D-3L and EQ-5D-5L scores.^23^

Unadjusted resource use, costs by resource category, total costs and QALYs are presented. Cost-effectiveness results were based on an adjusted analysis, controlling for a set of patient covariables and for potential imbalances in the number of revascularisations across arms. Patient covariables were selected based on multivariable p<0.1 and considered diabetes, family history of CHD, baseline ECG result, trial centre, body mass index, smoking status plus baseline EQ-5D in the QALY regression.^24^

Costs and QALYs were estimated within trial arm and pretest likelihood group conditional on whether a revascularisation was undertaken or not. Results are then calculated as the weighted average of revascularised and non-revascularised patients using common revascularisation rates within pretest likelihood groups. This assumes that any differences in revascularisation rates between trial arms reflect sampling error. Generalised linear models were used for the cost analysis, accounting for the non-normality and skewed nature of cost data.^25^ For QALYs an ordinary least squares regression was applied.

Results are presented in terms of mean costs and QALYs with 95% credible intervals. Incremental cost-effectiveness ratios (ICERs) were calculated, which report the incremental cost per QALY gained of one strategy compared with the next less effective and less costly strategy. A strategy was excluded from ICER calculations if it were strictly or extendedly dominated, that is, more costly and less effective or more costly per additional QALY than the next more effective strategy, respectively. Incremental net health benefits per patient of each strategy compared with NICE-guided care were also estimated, which capture the overall health gain from one individual receiving the care. This measure reflects any health benefits to that individual from the strategy plus/minus the impact on other individuals’ health from any change in resource requirements. If a strategy is cost-saving those resources can be used to improve the health of other patients, resulting in further health gain, while if it is more costly those resources are not available for others, resulting in health loss. Incremental net health benefits were estimated based on three measures of the QALYs that could have been generated elsewhere in the NHS from the same resources (health opportunity cost): £15 000 per QALY based on the Department of Health and Social Care’s chosen threshold,^26^ and £20 000 and £30 000 per QALY reflecting the range used by NICE.^9^

Uncertainty in these estimates and the probability of each strategy being the most costly, most effective and cost-effective (for each health opportunity cost) were estimated using Monte Carlo simulation. A number of scenario analyses were considered, including (1) not controlling for differential revascularisation rates across arms; (2) NHS tariff diagnostic costs; (3) diagnostic costs only; (4) revised MPS unit cost; (5) EQ-5D-5L scores; and (6) a pooled functional imaging strategy (CMR and MPS). Finally, we examined the unit cost of CMR at which it is no longer cost-effective.

## Results

### Resource use, costs and health outcomes

**Figure 2:**
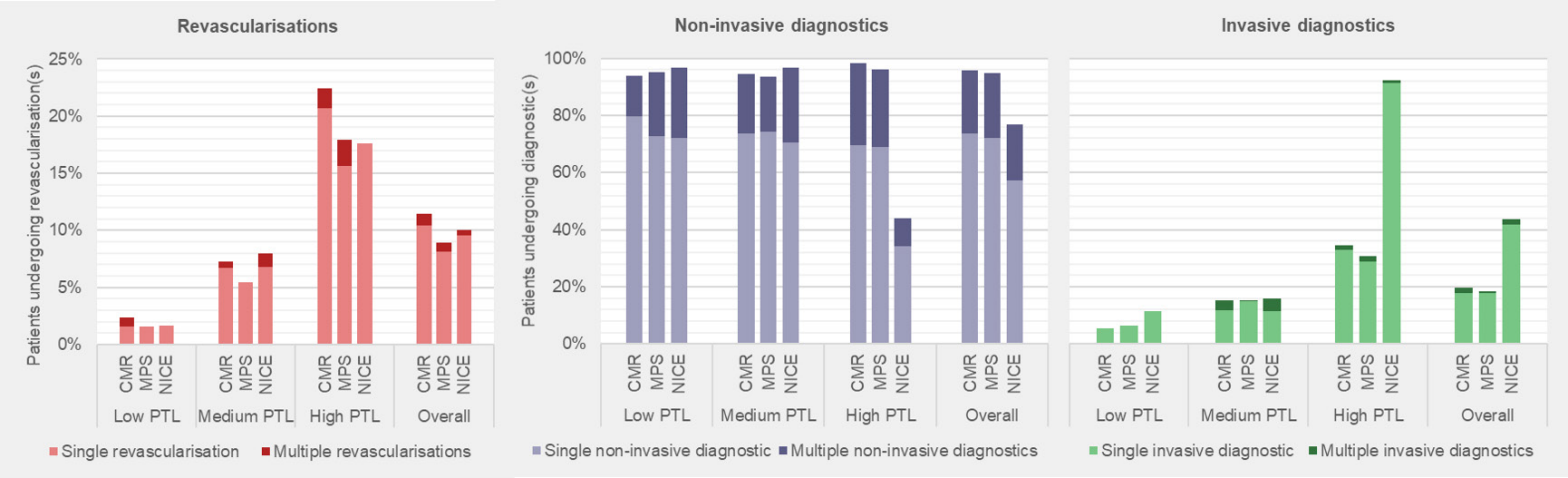
Proportion of patients who underwent diagnostic and revascularisation procedures. Non-invasive diagnostic is defined as any MPS, CMR, cardiac CT, exercise tolerance test, echocardiogram or stress echocardiogram. Revascularisation is defined by any percutaneous coronary intervention or coronary artery bypass grafting. CMR, cardiovascular magnetic resonance; MPS, myocardial perfusion scintigraphy; NICE, National Institute for Health and Care Excellence; PTL, pretest likelihood.

The average age of patients in the trial was 56.3 years, 46.9% of patients were women, and 26.1% were of low, 37.4% were of medium and 36.4% were of high pretest likelihood.^12^ Missing data were low for resource use but higher for EQ-5D scores. Table 2 presents unadjusted diagnostic, revascularisation and total costs, along with annual and total QALYs. Figure 2 presents the proportion of patients who underwent diagnostic and revascularisation procedures. Online supplementary tables S3–S6 report further costs, resource use and EQ-5D scores.

**Table 2:** Estimated mean costs and QALYs per patient (unadjusted)

	CMR (n=481)				MPS (n=481)				NICE (n=240)			
	LPTL	MPTL	HPTL	Overall	LPTL	MPTL	HPTL	Overall	LPTL	MPTL	HPTL	Overall
Mean costs*	**n=128**	**n=179**	**n=174**	**n=481**	**n=125**	**n=183**	**n=173**	**n=481**	**n=61**	**n=88**	**n=91**	**n=240**
Diagnostic cost (SD)	£465.59 (360)	£595.22 (548)	£849.64 (620)	£652.76 (556)	£676.62 (298)	£761.23 (500)	£967.91 (616)	£813.58 (518)	£446.05 (417)	£831.24 (614)	£1122.27 (309)	£843.69 (535)
Revascularisation cost (SD)	£165.16 (1137)	£420.43 (1548)	£1271.66 (2464)	£660.43 (1908)	£100.90 (899)	£285.91 (1193)	£1083.67 (2491)	£524.76 (1765)	£86.64 (677)	£472.42 (1703)	£929.26 (2023)	£547.59 (1680)
Other costs† (SD)	£231.33 (891)	£335.23 (757)	£619.96 (1014)	£410.58 (905)	£260.90 (555)	£300.22 (513)	£485.99 (680)	£356.82 (595)	£224.54 (225)	£353.40 (600)	£413.65 (460)	£343.49 (498)
Total cost (SD)	£862.08 (2192)	£1350.89 (2294)	£2741.26 (3392)	£1723.77 (2828)	£1038.42 (1357)	£1347.37 (1777)	£2537.57 (3171)	£1695.15 (2375)	£757.24 (1052)	£1657.06 (2632)	£2465.18 (2218)	£1734.77 (2259)
Mean QALYs*	**n=128**	**n=179**	**n=174**	**n=481**	**n=125**	**n=183**	**n=173**	**n=481**	**n=61**	**n=88**	**n=91**	**n=240**
Year 1 (SD)	0.7965 (0.2380)	0.7700 (0.2508)	0.7374 (0.2593)	0.7652 (0.2513)	0.7687 (0.2351)	0.7502 (0.2611)	0.7384 (0.2340)	0.7508 (0.2487)	0.7239 (0.2519)	0.7016 (0.2595)	0.7388 (0.2359)	0.7214 (0.2489)
Year 2 (SD)	0.7800 (0.2585)	0.7434 (0.3101)	0.7306 (0.2925)	0.7485 (0.3101)	0.7499 (0.2387)	0.7369 (0.3092)	0.7074 (0.2737)	0.7296 (0.3001)	0.7096 (0.2816)	0.6581 (0.2953)	0.6983 (0.3013)	0.6864 (0.2883)
Year 3 (SD)	0.7503 (0.2496)	0.7210 (0.2805)	0.6965 (0.3028)	0.7200 (0.2937)	0.7169 (0.2442)	0.7076 (0.3070)	0.6777 (0.2723)	0.6993 (0.2918)	0.6844 (0.2754)	0.6437 (0.2670)	0.6689 (0.3070)	0.6636 (0.2681)
Total QALYs (SD)	2.3268 (0.6879)	2.2344 (0.7954)	2.1645 (0.8108)	2.2337 (0.8146)	2.2355 (0.6592)	2.1948 (0.8299)	2.1234 (0.7383)	2.1797 (0.7939)	2.1179 (0.7606)	2.0034 (0.7737)	2.1060 (0.7878)	2.0714 (0.7515)

*Discounted at 3.5% rate per annum

†Other costs comprise cardiovascular-related inpatient, outpatient and drug-related costs.

CMR, cardiovascular magnetic resonance; HPTL, high pretest likelihood; LPTL, low pretest likelihood; MPS, myocardial perfusion scintigraphy; MPTL, medium pretest likelihood; NICE, National Institute for Health and Care Excellence guidance; QALY, quality-adjusted life-years.

Overall, patients in the NICE arm were most likely to have received ICA (43.8%). Revascularisation rates were comparable but highest in the CMR arm (11.4%). Total costs were similar between the arms (£1695–£1735), with CMR’s lower diagnostic costs offset by higher revascularisation costs. Mean QALYs were lowest in the NICE arm and highest in the CMR arm.

In low pretest likelihood patients, a higher proportion in the NICE arm underwent ICA (11.5%) than in the CMR (5.5%) and MPS (6.4%) arms, and only a small proportion of patients received a revascularisation procedure (1.6%–2.3%). The MPS arm had the highest total mean cost at £1038, compared with £862 and £757 in the CMR and NICE arms, respectively. Mean QALYs were lowest in the NICE arm and highest in the CMR arm.

For medium pretest likelihood patients, similar proportions of patients underwent ICA (15.1%–15.9%) across the trial arms, although revascularisations were lowest for MPS. Revascularisation costs were lowest for the MPS arm, while diagnostic costs were highest for the NICE arm. NICE arm had the highest total mean cost at £1657, while CMR (£1351) and MPS (£1347) incurred comparable costs. Mean QALYs were similar between CMR and MPS, but markedly lower in the NICE arm.

In high pretest likelihood patients, the NICE arm had the highest proportion of patients receiving ICA (92.3%), although this was below the 100% protocol for NICE guidelines. Revascularisation rates and mean total costs were highest for CMR, with a mean cost totalling £2741, compared with £2538 and £2465 in the MPS and NICE arms, respectively. Mean QALYs were highest in the CMR arm, although similar across the trial arms.

### Cost-effectiveness

**Figure 3:**
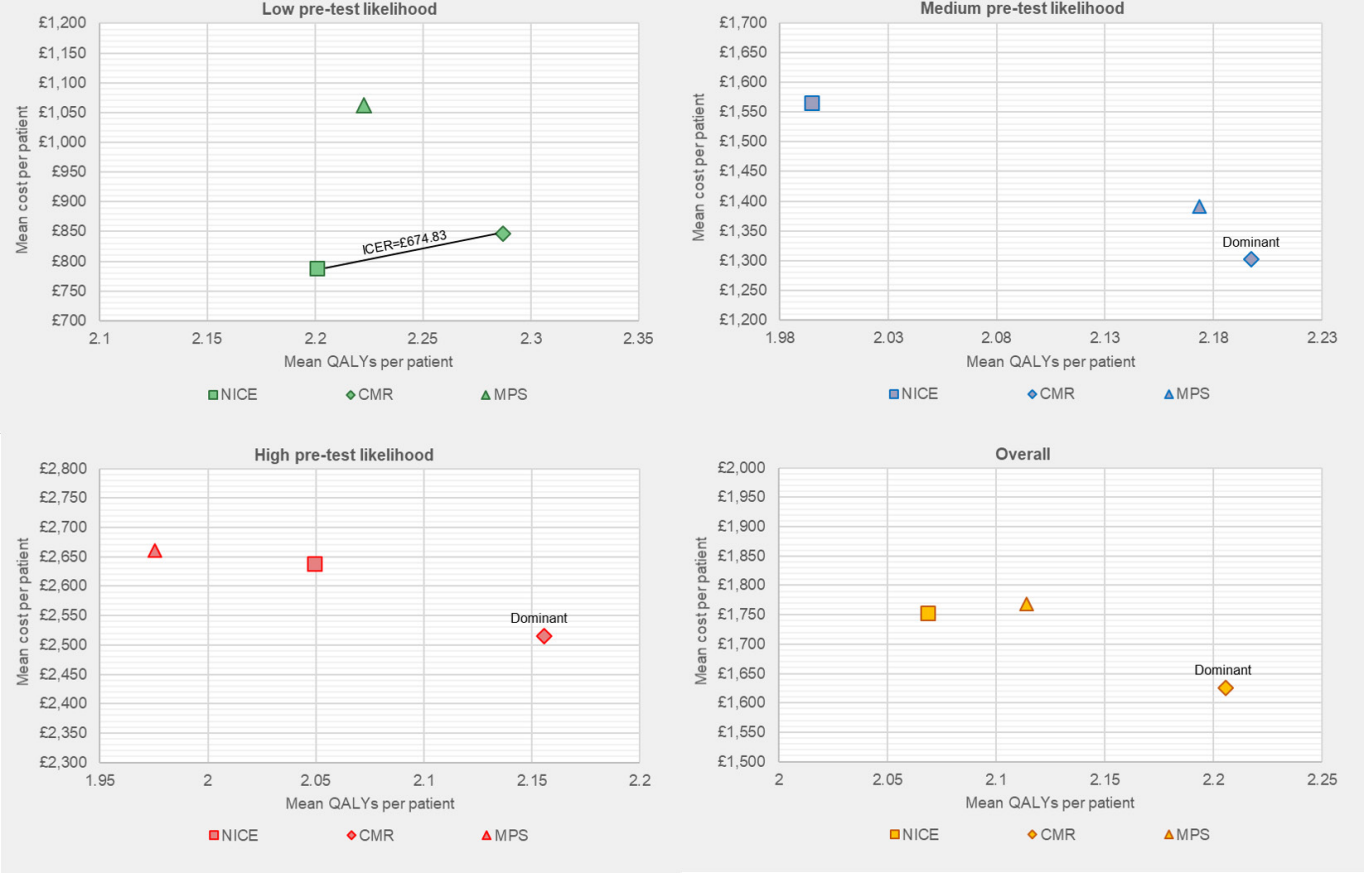
Base-case cost-effectiveness plane. CMR, cardiovascular magnetic resonance; ICER, incremental cost-effectiveness ratio; MPS, myocardial perfusion scintigraphy; NICE, National Institute for Health and Care Excellence guidance; QALY, quality-adjusted life-years.

Table 3 presents the adjusted base-case mean costs, QALYs and cost-effectiveness results for each trial arm overall and by pretest likelihood stratum. Figure 3 presents the results on cost-effectiveness planes. Online supplementary figure S1 presents the cost-effectiveness acceptability curves.

**Table 3:** Base-case cost-effectiveness results

	Mean cost per patient	Mean QALY per patient	ICER	Incremental mean net health benefit per patient* (95% CI)		
	(95% CI)	(95% CI)		k=£15 000	k=£20 000	k=£30 000
	(P (most costly))	(P (most effective))		(probability of being cost-effective)		
Low PTL						
NICE	£787.92	2.20095		–	–	–
	(531.64 to 1046.85)	(2.05635 to 2.36087)		–	–	–
	(0.047)	(0.130)		(0.160)	(0.153)	(0.147)
CMR	£846.00	2.28702	£674.83	0.082	0.083	0.084
	(597.5 to 1098.28)	(2.19106 to 2.38562)		(−0.096 to 0.257)	(−0.093 to 0.258)	(−0.091 to 0.259)
	(0.083)	(0.719)		(0.736)	(0.729)	(0.722)
MPS	£1061.94	2.22261	Dominated	0.003	0.008	0.013
	(835.89 to 1293.69)	(2.13167 to 2.3122)		(−0.173 to 0.171)	(−0.166 to 0.173)	(−0.161 to 0.177)
	(0.870)	(0.151)		(0.104)	(0.118)	(0.131)
Medium PTL						
CMR	£1301.97	2.19738		0.220	0.216	0.211
	(1056.23 to 1560.61)	(2.09621 to 2.29582)		(0.047 to 0.398)	(0.041 to 0.393)	(0.036 to 0.388)
	(0.074)	(0.642)		(0.688)	(0.682)	(0.671)
MPS	£1391.20	2.17327	Dominated	0.19	0.187	0.184
	(1105.68 to 1684.8)	(2.0717 to 2.26984)		(0.023 to 0.36)	(0.021 to 0.355)	(0.018 to 0.355)
	(0.211)	(0.353)		(0.310)	(0.316)	(0.326)
NICE	£1565.28	1.99474	Dominated	–	–	–
	(1114.65 to 1991.53)	(1.85789 to 2.12644)		–	–	–
	(0.715)	(0.005)		(0.002)	(0.002)	(0.003)
High PTL						
CMR	£2514.86	2.15591		0.115	0.113	0.111
	(2058.12 to 2936.73)	(2.06893 to 2.24084)		(−0.18 to 0.406)	(−0.185 to 0.401)	(−0.186 to 0.395)
	(0.184)	(0.747)		(0.758)	(0.755)	(0.750)
NICE	£2638.37	2.04944	Dominated	–	–	–
	(2048.84 to 3213.82)	(1.78434 to 2.3057)		–	–	–
	(0.400)	(0.213)		(0.204)	(0.207)	(0.211)
MPS	£2660.93	1.97525	Dominated	−0.076	−0.075	−0.075
	(2154.92 to 3139.48)	(1.76637 to 2.18188)		(−0.368 to 0.213)	(−0.361 to 0.212)	(−0.358 to 0.213)
	(0.416)	(0.040)		(0.038)	(0.038)	(0.039)
Overall						
CMR	£1624.82	2.20568		0.146	0.144	0.141
	(1431.4 to 1824.44)	(2.14564 to 2.26468)		(−0.013 to 0.306)	(−0.013 to 0.302)	(−0.013 to 0.302)
	(0.073)	(0.917)		(0.931)	(0.926)	(0.925)
NICE	£1753.24	2.06854	Dominated	–	–	–
	(1473.15 to 2031.75)	(1.92352 to 2.20276)		–	–	–
	(0.440)	(0.035)		(0.029)	(0.032)	(0.031)
MPS	£1767.87	2.11400	Dominated	0.044	0.045	0.045
	(1571.78 to 1989.15)	(2.01256 to 2.2169)		(−0.103 to 0.189)	(−0.101 to 0.187)	(−0.1 to 0.187)
	(0.487)	(0.048)		(0.040)	(0.042)	(0.044)

P (most costly): probability of a strategy being the most costly alternative.

P (most effective): probability of a strategy being the most effective alternative (ie, highest QALY gain).

*All incremental net health benefits are estimated compared with NICE-guided care.

CI, credible intervals; CMR, cardiovascular magnetic resonance; ICER, incremental cost-effectiveness ratio; k, cost-effectiveness threshold; MPS, myocardial perfusion scintigraphy; NICE, National Institute for Health and Care Excellence guidance; PTL, pretest likelihood; QALY, quality-adjusted life-years.

Overall, CMR was found to be the least costly and most effective strategy, with a 7% probability of being most costly and a 92% probability of the highest QALY gain. The mean incremental net health benefit per patient of CMR compared with NICE ranged from 0.141 to 0.146 QALYs across the health opportunity cost estimates considered, and the probability of CMR being cost-effective is over 93% for each. However, there is considerable uncertainty with the 95% credible intervals across all three arms overlapping for mean costs and QALYs.

In low pretest likelihood patients, the NICE arm was the least costly and least effective strategy. CMR was the second most costly and the most effective, with an ICER of £675 per QALY and a mean incremental net health benefit ranging from 0.082 to 0.084 QALYs per patient. The probability of CMR being cost-effective exceeded 72% for each health opportunity cost estimate considered.

For medium pretest likelihood patients, the CMR arm was the least costly and most effective strategy, while NICE was the least effective and most costly option. CMR had a mean incremental net health benefit ranging from 0.211 to 0.220 QALYs per patient, with the probability of CMR being cost-effective exceeding 67% for each health opportunity cost estimate considered.

For high pretest likelihood patients, the CMR arm was the least costly and most effective strategy, whereas MPS was the least effective and most costly option, and NICE the second most costly and the second most effective. CMR had mean incremental net health benefit ranging from 0.111 to 0.115 QALYs per patient, with the probability of CMR being cost-effective exceeding 75% for each health opportunity cost estimate considered.

### Scenario analysis

Table 4 presents the cost-effectiveness results for the scenario analyses. Online supplementary tables S7–S13 present the detailed breakdown of these results.

When not controlling for differential revascularisation rates across arms, conclusions remain unchanged, although with results comparably less favourable for CMR.

**Table 4:** Scenario analyses: cost-effectiveness results

		Mean cost per patient	Mean QALY per patient	ICER			Mean cost per patient	Mean QALY per patient	ICER
Not controlling for differential revascularisation rates					NHS tariffs				
Low PTL	NICE	£739.72	2.20158		Low PTL	NICE	£727.67	2.20095	
	CMR	£890.25	2.27686	£1999.70		MPS	£791.10	2.22261	Dominated
	MPS	£1066.30	2.22813	Dominated		CMR	£875.51	2.28702	£1310.57
Medium PTL	CMR	£1314.13	2.21530		Medium PTL	MPS	£1135.14	2.17327	
	MPS	£1322.85	2.23666	Dominated		NICE	£1309.26	1.99474	Dominated
	NICE	£1619.41	2.05109	Dominated		CMR	£1311.50	2.19738	£7317.38
High PTL	NICE	£2484.19	2.17658		High PTL	MPS	£2347.23	1.97525	
	MPS	£2504.96	2.14166	Dominated		CMR	£2532.14	2.15591	£1023.51
	CMR	£2728.52	2.19613	£12 495.02		NICE	£2604.59	2.04944	Dominated
Overall	MPS	£1686.59	2.19981		Overall	MPS	£1486.94	2.11400	
	NICE	£1704.73	2.13613	Dominated		NICE	£1629.34	2.06854	Dominated
	CMR	£1718.79	2.22440	£1309.99		CMR	£1642.40	2.20568	£1695.61
Diagnostic costs only					Revised MPS unit costs				
Low PTL	NICE	£430.21	2.20095		Low PTL	NICE	£775.50	2.20095	
	CMR	£458.95	2.28702	£333.92		MPS	£806.95	2.22261	Extendedly dominated
	MPS	£695.64	2.22261	Dominated		CMR	£844.72	2.28702	£586.35
Medium PTL	CMR	£581.88	2.19738		Medium PTL	MPS	£1143.37	2.17327	
	MPS	£768.27	2.17327	Dominated		CMR	£1308.54	2.19738	£6853.24
	NICE	£821.61	1.99474	Dominated		NICE	£1313.72	1.99474	Dominated
High PTL	CMR	£827.45	2.15591		High PTL	MPS	£2398.76	1.97525	
	MPS	£988.42	1.97525	Dominated		CMR	£2511.51	2.15591	£768.46
	NICE	£1 1123.81	2.04944	Dominated		NICE	£2621.23	2.04944	Dominated
Overall	CMR	£639.25	2.20568		Overall	MPS	£1512.94	2.11400	
	NICE	£829.48	2.06854	Dominated		CMR	£1625.73	2.20568	£1230.20
	MPS	£829.52	2.11400	Dominated		NICE	£1649.57	2.06854	Dominated
EQ-5D-5L						PFIA			
Low PTL	NICE	£787.92	2.43234		Low PTL	NICE	£794.34	2.15534	
	CMR	£846.00	2.47754	£1285.02		PFIA	£963.27	2.25924	£1625.92
	MPS	£1061.94	2.45839	Dominated					
Medium PTL	CMR	£1301.97	2.41772		Medium PTL	PFIA	£1344.22	2.20270	
	MPS	£1391.20	2.40701	Dominated		NICE	£1563.73	2.02996	Dominated
	NICE	£1565.28	2.30330	Dominated					
High PTL	CMR	£2514.86	2.36506		High PTL	PFIA	£2579.61	2.13792	
	NICE	£2638.37	2.26125	Dominated		NICE	£2617.76	2.14353	£6800.50
	MPS	£2660.93	2.31846	Dominated					
Overall	CMR	£1624.82	2.41415		Overall	PFIA	£1695.81	2.19386	
	NICE	£1753.24	2.32169	Dominated		NICE	£1747.78	2.10410	Dominated
	MPS	£1767.87	2.38816	Dominated					

The full results for each scenario are reported in online supplementary tables S7–S12.

CMR, cardiovascular magnetic resonance; ICER, incremental cost-effectiveness ratio; MPS, myocardial perfusion scintigraphy; NHS, National Health Service; NICE, National Institute for Health and Care Excellence guidance; PFIA, pooled functional imaging arm; PTL, pretest likelihood; QALY, quality-adjusted life-years.

When using tariff-based diagnostic unit costs, CMR became most costly overall and for the low and medium pretest likelihood groups. Overall and for each pretest likelihood stratum, CMR had positive incremental net health benefits and ICERs below the health opportunity cost estimates considered.

Considering diagnostic costs only made the cost differentials more favourable for CMR overall and for each pretest likelihood stratum.

With revised MPS costs, MPS becomes the least costly arm overall and for the medium and high pretest likelihood strata. Overall and for each pretest likelihood stratum, CMR had positive incremental net health benefits and ICERs below the health opportunity cost estimates considered.

Using EQ-5D-5L scores to estimate QALYs did not have a marked impact on the cost-effectiveness results, with conclusions remaining unchanged. For all patients, QALYs are notably higher with EQ-5D-5L, reflecting differences between the EQ-5D-3L and EQ-5D-5L value sets.

The pooled functional imaging arm was found to be more effective overall and in the low and medium pretest likelihood strata, and less costly in the medium and high pretest likelihood strata. Pooled functional imaging was found to be cost-effective overall (dominant) and for low pretest likelihood (ICER of £1626 per QALY) and medium pretest likelihood patients (dominant). For high pretest likelihood patients, NICE was more expensive and more effective with an ICER of £6801 per QALY (cost-effective at health opportunity cost estimates considered).

Finally, we considered what the cost of CMR would have to be for the CMR to not be cost-effective at the different health opportunity costs (online supplementary table S13). The costs would have to be markedly higher than the £394 unit cost (minimum £938, maximum £4129) for CMR not to be cost-effective.

## Discussion

This study has estimated the resource use, costs and cost-effectiveness of diagnostic strategies for patients with suspected CHD based on the CE-MARC 2 trial. Controlling for revascularisation status and patients’ baseline characteristics, CMR was determined to be the most effective and cost-effective strategy overall and for all pretest likelihood subgroups. The results remained robust across alternative scenarios, despite the differences in costs and outcomes being highly uncertain.

These findings need to be interpreted with caution. Results are potentially driven by random baseline imbalances between the arms, where smaller numbers of patients, with potentially different baseline prognoses and care requirements, inform each strategy and pretest likelihood stratum. The potential impact of random baseline imbalances is demonstrated with the MPS and NICE arms being equivalent strategies in the medium pretest likelihood group (ie, both strategies scheduling MPS as an initial test), but the latter incurring more costs (£174) and associated with less QALYs (−0.1785). Furthermore, not controlling for differential revascularisation rates across trial arms provides less favourable results for CMR due to what may have been random imbalances in patients requiring revascularisation. Expanding the analysis to consider more types of costs does incorporate potentially relevant observed differentials in resource use, but also increases the potential for random imbalances impacting results. Nonetheless, our conclusions remained robust when considering only the costs of the diagnostic procedures.

The current findings suggest that CMR directed care is cost-effective and has a high prognostic value relative to MPS and NICE guidelines directed care. These findings support a previous analysis of the CE-MARC study,^5^ but conflict with those of Genders *et al*,^6^ who found that CCT was likely to be cost-effective in low-risk patients, a strategy akin to the NICE guidelines arm, which was the least effective arm in low pretest likelihood patients and deemed not cost-effective. Our results inform a knowledge gap relating to the cost-effectiveness of CCT-guided management. The results of the SCOT-HEART trial have informed an update of the NICE CG95 guidelines.^8 10^ However, in SCOT-HEART, compared with standard care, the CCT strategy was associated with more angina and worse quality of life.^27^ A health economic analysis of the SCOT-HEART trial has not been reported.

This study has a number of strengths and weaknesses. The analysis is based on a large and pragmatic diagnostic strategy study which has the potential to demonstrate the impact of each strategy on patients’ costs and outcomes. By controlling for patient covariables and the impact of revascularisation status, attempts have been made to address what may be considered baseline imbalances. However, there are a number of weaknesses. First, it is difficult for diagnostics to influence downstream costs and benefits, other than through their impact on treatment decisions, and whether long-term differences in observed costs and outcomes in the trial are attributable to the alternative diagnostic strategies is uncertain. Second, estimating results conditional on revascularisation status generates more imprecise estimates, particularly in the low pretest likelihood group where revascularisation was rarely performed. Third, we have not considered the implications beyond 3 years, for example, any subsequent impacts on mortality or morbidity or the impact of ionising radiation on cancer incidence. Fourth, we have only considered the strategies included in the CE-MARC 2 trial and this excluded other potentially relevant comparators which may be cost-effective.^6 8^ Fifth, while recent trials such as ISCHEMIA may suggest diagnostic imaging could be used more judiciously, with ageing populations and increasing multimorbidity it is likely overall rates of imaging will continue to increase.^28^ Sixth, the analysis has been undertaken from the NHS perspective and only considers costs and benefits relevant to the NHS; a broader ‘societal’ perspective may consider other impacts such as productivity and environmental costs.^29^ Lastly, the potential for random baseline imbalances remains, while the results themselves are highly uncertain.

In conclusion, this study has estimated the costs and cost-effectiveness of diagnostic strategies. The results suggest CMR is cost-effective overall and across pretest likelihood subgroups, compared with MPS and NICE guidelines-guided care.

**Key messages**

**What is already known on this subject?**

- The optimal strategy for the diagnosis of patients with suspected coronary heart disease is a source of much debate.
- Despite the availability of non-invasive imaging, invasive coronary angiography remains frequently used despite its higher cost and associated risks.

**What might this study add?**

- This study estimated the resource use, costs and cost-effectiveness of different diagnostic strategies for patients with suspected coronary heart disease based on the Clinical Evaluation of Magnetic Resonance Imaging in Coronary Heart Disease 2 trial.
- Cardiovascular magnetic resonance-guided care was determined to be the most effective and cost-effective strategy overall and for all pretest likelihood subgroups.
- The results remained robust across various alternative scenarios, despite the differences in costs and outcomes between each strategy being highly uncertain.

**How might this impact on clinical practice?**

- The findings of this study support the expanded use of cardiovascular magnetic resonance-guided care in the diagnosis of patients with suspected coronary heart disease.
- The study suggests that this can improve health outcomes while lowering costs.
