# Supplementary material for: Cost-effectiveness of cardiovascular imaging for stable coronary heart disease
Source: Heart. 2020 Aug 14;107(5):381–8. doi: 10.1136/heartjnl-2020-316990 (PMC7892375; doi:10.1136/heartjnl-2020-316990)
Supplement: Supplementary data [file heartjnl-2020-316990supp001.pdf]

Supplementary material

Table S1- Unit costs for additional resource use to those detailed in Table 1 [13–16]

	Unit Cost	Source
**Hospitalisations**		
MI	£2,213.05	NHS Reference Costs 2016/17
Stroke	£4,205.34	NHS Reference Costs 2016/17
Arrhythmia	£1,837.33	NHS Reference Costs 2016/17
Heart Failure	£1,695.97	NHS Reference Costs 2016/17
Chest pain	£1,238.63	NHS Reference Costs 2016/17
Serious adverse event	£2,084.91	NHS Reference Costs 2016/17
Day in CCU	£1,301.23	NHS Reference Costs 2016/17
Day in HDU	£572.23	NHS Reference Costs 2016/17
Day in ICU	£1,301.23	NHS Reference Costs 2016/17
Day case	£738.05	NHS Reference Costs 2016/17
General Ward	£364.86	NHS Reference Costs 2016/17
Clinical Decision Unit	£364.86	NHS Reference Costs 2016/17
**Outpatient appointments**		
Cardiology & Cardiothoracic	£128.73	NHS Reference Costs 2016/17
**Cardiovascular medication**		
All	Various	British National Formulary and Drugs and Pharmaceuticals Electronic Market Information Database

CCU: Critical care unit; HDU: High dependency unit; ICU: Intensive care unit; MI: Myocardial infarction

Table S2- Alternative unit costs as used in the scenario analysis in Table 4 [13,16]

Diagnostic Test Unit Costs	Unit Cost	Source
**NHS Tariffs**		
Cardiac Computed Tomography	£206.00	NHS Tariff 2016/17
Cardiovascular magnetic resonance	£426.00	NHS Tariff 2016/17
Myocardial Perfusion Scintigraphy	£323	NHS Tariff 2016/17
Coronary Angiography	£1,061	NHS Tariff 2016/17
**Revised MPS cost**		
Myocardial Perfusion Scintigraphy	£325.75	NHS Reference Costs 2016/17

Table S3: Total number of diagnostic procedures and revascularisations over time

	CMR (n=481)						MPS (n=481)						NICE (n=240)					
	Year 1						Year 1						Year 1					
	90d	90-365	0-365	Year 2	Year 3	Total	90d	90-365	0-365	Year 2	Year 3	Total	90d	90-365	0-365	Year 2	Year 3	Total
**Low PTL**	n=128						n=125						n=61					
CCT																		
patients	0	0	0	1	0	1	1	4	5	1	1	6	52	3	55	0	0	55
total	0	0	0	1	0	1	1	4	5	1	1	7	52	3	55	0	0	55
MPS																		
patients	1	1	2	2	1	4	115	3	118	1	0	119	2	1	3	0	0	3
total	1	1	2	2	1	5	115	3	118	1	0	119	2	1	3	0	0	3
CMR																		
patients	108	8	116	0	0	116	0	0	0	0	0	0	0	1	1	0	1	2
total	108	8	116	0	0	116	0	0	0	0	0	0	0	1	1	0	1	2
CA																		
patients	4	3	7	0	0	7	4	4	8	0	0	8	2	5	7	0	0	7
total	4	3	7	0	0	7	4	4	8	0	0	8	2	5	7	0	0	7
ETT																		
patients	2	2	4	4	2	8	1	1	2	2	2	6	0	3	3	1	0	4
total	2	2	4	4	2	10	1	1	2	2	2	6	0	3	3	1	0	4
ECHO																		
patients	3	4	7	4	1	12	6	8	14	0	4	18	3	3	6	3	3	12
total	3	4	7	5	1	13	6	8	14	0	4	18	3	3	6	3	3	12
sECHO																		
patients	0	1	1	0	1	2	2	1	3	0	1	4	0	2	2	0	1	3
total	0	1	1	0	1	2	2	1	3	0	1	4	0	2	2	0	1	3
PCI																		
patients	2	2	3	0	0	3	0	1	1	0	0	1	0	0	0	0	0	0
total	2	2	4	0	0	4	0	1	1	0	0	1	0	0	0	0	0	0
CABG																		
patients	0	0	0	0	0	0	0	1	1	0	0	1	1	0	1	0	0	1
total	0	0	0	0	0	0	0	1	1	0	0	1	1	0	1	0	0	1

BMJ Publishing Group Limited (BMJ) disclaims all liability and responsibility arising from any reliance placed on this supplemental material which has been supplied by the author(s)

**Med PTL**	**n=179**						**n=183**						**n=88**					
CCT																		
patients	0	5	5	0	3	8	1	4	5	3	2	10	0	1	1	0	0	1
total	0	5	5	0	3	8	1	4	5	3	2	10	0	1	1	0	0	1
MPS																		
patients	1	0	1	0	0	1	160	6	165	0	0	165	82	1	83	0	0	83
total	1	0	1	0	0	1	160	6	166	0	0	166	82	1	83	0	0	83
CMR																		
patients	145	12	157	0	0	157	2	2	3	4	1	8	0	2	2	1	1	3
total	145	12	157	0	0	157	2	2	4	4	1	9	0	2	2	1	1	4
CA																		
patients	14	8	22	5	2	27	4	16	20	3	5	28	3	8	11	2	4	14
total	14	11	25	5	3	33	4	16	20	4	5	29	3	9	12	2	4	18
ETT																		
patients							2	4	6	1	2	9	1	2	3	1	3	6
total	3	8	11	9	0	20	2	4	6	1	2	9	1	2	3	1	3	7
ECHO																		
patients	6	4	10	6	5	21	10	10	20	3	2	25	3	6	9	1	6	16
total	6	4	10	6	5	21	10	10	20	3	2	25	3	6	9	1	6	16
sECHO																		
patients	4	0	4	1	0	5	1	0	1	1	0	2	1	1	2	0	1	3
total	4	0	4	1	0	5	1	0	1	1	0	2	1	1	2	0	1	3
PCI*																		
patients	7	3	10	1	1	12	1	5	6	1	1	8	0	3	3	2	1	6
total	7	3	10	1	1	12	1	5	6	1	1	8	0	4	4	2	1	7
CABG*																		
patients	0	1	1	1	0	2	2	0	2	0	0	2	0	1	1	0	0	1
total	0	1	1	1	0	2	2	0	2	0	0	2	0	1	1	0	0	1
**High PTL**	**n=174**						**n=173**						**n=91**					
CCT																		
patients	0	3	3	3	2	7	0	2	2	0	0	2	0	0	0	0	1	1
total	0	3	3	3	2	8	0	2	2	0	0	2	0	0	0	0	1	1

MPS																		
patients	3	2	5	1	0	6	162	0	162	3	0	164	2	2	4	3	0	7
total	3	2	5	1	0	6	162	0	162	3	0	165	2	2	4	3	0	7
CMR																		
patients	160	2	162	2	2	163	0	4	4	0	1	5	5	0	5	0	1	6
total	160	2	162	2	2	166	0	4	4	0	1	5	5	0	5	0	1	6
CA																		
patients	37	20	57	3	3	60	16	34	50	4	1	53	79	5	84	0	0	84
total	38	20	58	4	3	65	16	35	51	5	1	57	80	5	85	0	0	85
ETT																		
patients	5	6	11	3	5	17	3	1	4	2	4	10	2	1	3	1	0	4
total	5	6	11	3	5	19	3	1	4	2	4	10	2	1	3	1	0	4
ECHO																		
patients	5	12	17	9	8	34	7	13	20	9	9	38	15	8	23	4	0	27
total	5	12	17	9	9	35	7	14	21	9	9	39	15	8	23	4	0	27
sECHO																		
patients	2	1	3	0	1	4	0	0	0	1	0	1	1	1	2	1	1	4
total	2	1	3	0	1	4	0	0	0	1	0	1	1	1	2	1	1	4
PCI†																		
patients	17	8	24	2	1	26	5	16	21	2	0	22	7	4	11	0	0	11
total	17	8	25	2	1	28	6	16	22	3	0	25	7	4	11	0	0	11
CABG†																		
patients	1	13	14	0	0	14	1	9	10	0	0	10	1	4	5	0	0	5
total	1	13	14	0	0	14	1	9	10	0	0	10	1	4	5	0	0	5
**Overall**	**n=481**						**n=481**						**n=240**					
CCT																		
patients	0	8	8	4	5	15	2	10	12	4	3	18	52	4	56	0	1	57
total	0	8	8	4	5	17	2	10	12	4	3	19	52	4	56	0	1	57
MPS																		
patients	5	3	7	3	1	11	437	9	445	4	0	448	86	4	90	3	0	93
total	5	3	8	3	1	12	437	9	446	4	0	450	86	4	90	3	0	93
CMR																		

patients	413	22	435	2	2	436	1	6	7	4	2	13	5	3	8	1	3	11
total	413	22	435	2	2	439	2	6	8	4	2	14	5	3	8	1	3	12
CA																		
patients	55	31	86	8	5	94	24	54	78	7	6	89	84	18	102	2	4	105
total	56	34	90	9	6	105	24	55	79	9	6	94	85	19	104	2	4	110
ETT																		
patients	10	16	26	16	7	42	6	6	12	5	8	25	3	6	9	3	3	14
total	10	16	26	16	7	49	6	6	12	5	8	25	3	6	9	3	3	15
ECHO																		
patients	14	20	34	19	14	67	23	31	54	12	15	81	21	17	38	8	9	55
total	14	20	34	20	15	69	23	32	55	12	15	82	21	17	38	8	9	55
sECHO																		
patients	6	2	8	1	2	11	3	1	4	2	1	7	2	4	6	1	3	10
total	6	2	8	1	2	11	3	1	4	2	1	7	2	4	6	1	3	10
PCI																		
patients	26	13	37	3	2	41	6	22	28	3	1	31	7	7	14	2	1	17
total	26	13	39	3	2	44	7	22	29	4	1	34	7	8	15	2	1	18
CABG																		
patients	1	14	15	1	0	16	3	10	13	0	0	13	2	5	7	0	0	7
total	1	14	15	1	0	16	3	10	13	0	0	13	2	5	7	0	0	7

* One patient randomised to CMR with a medium PTL had both a CABG and a PCI procedure meaning only 13 patients in total received a revascularisation

† Three patients randomised to CMR with a high PTL had two revascularisation procedures (two patients having 2 PCI’s and one patient having a CABG and PCI) meaning only 39 patients received a revascularisation; four patients randomised to MPS with a high PTL had two revascularisation procedures (3 patients having 2 PCI’s and one patient having a CABG and PCI) meaning only 31 patients received a revascularisation

LPTL: Low pre-test likelihood; MPTL: Medium pre-test likelihood; HPTL: High pre-test likelihood; CMR: Cardiovascular Magnetic Resonance; MPS: Myocardial Perfusion Scintigraphy; NICE: National Institute for Health and Social Care Excellence Guidance; CTT: Cardiac Computed Tomography; CA: Coronary Angiography; ETT: Exercise Tolerance Test; ECHO: Echocardiogram; sECHO: Stress echocardiogram ; PCI: Percutaneous Coronary Intervention ; CABG: Coronary Artery Bypass Graft

BMJ Publishing Group Limited (BMJ) disclaims all liability and responsibility arising from any reliance placed on this supplemental material which has been supplied by the author(s)

Table S4: Mean costs per patient by resource category (discounted at 3.5% per annum)

	CMR (n=481)					
	Year 1					
	90d	90-365	0-365	Year 2	Year 3	Total†
**LPTL**	n=128					
Diag (SD)	£375.50 (227)	£61.65 (194)	£437.15 (294)	£20.15 (99)	£8.29 (56)	£465.59 (360)
Revas (SD)	£82.58 (658)	£82.58 (658)	£165.16 (1137)	0 (0)	0 (0)	£165.16 (1137)
Inpatient* (SD)	£43.38 (347)	£48.38 (451)	£91.77 (716)	£0.09 (0.65)	£0.05 (0.37)	£91.90 (717)
Outpatient (SD)	£44.25 (63)	£21.12 (68)	£65.37 (96)	£12.27 (57)	£12.07 (50)	£88.49 (141)
Drug (SD)	£5.18 (11)	£13.51 (27)	£18.70 (34)	£17.13 (46)	£16.81 (37)	£50.94 (105)
Total (SD)	£550.90 (996)	£227.25 (1155)	£778.15 (1986)	£48.64 (178)	£35.29 (103)	£862.08 (2192)
**MPTL**	n=179					
Diag (SD)	£415.59 (338)	£108.68 (335)	£524.27 (450)	£41.32 (193)	£29.63 (227)	£595.22 (548)
Revas (SD)	£206.68 (1027)	£118.10 (783)	£324.79 (1391)	£59.05 (557)	£41.34 (523)	£420.43 (1548)
Inpatient* (SD)	£60.09 (314)	£25.49 (203)	£85.57 (430)	£0.04 (0.23)	£0.01 (0.11)	£85.62 (430)
Outpatient (SD)	£39.55 (87)	£65.44 (236)	£105.00 (304)	£30.64 (77)	£16.11 (58)	£149.64 (351)
Drug (SD)	£6.01 (7)	£25.43 (67)	£31.44 (72)	£35.87 (106)	£36.28 (89)	£99.97 (251)
Total (SD)	£727.93 (1381)	£343.14 (1132)	£1071.07 (1946)	£162.68 (706)	£117.14 (749)	£1350.89 (2294)
**HPTL**	n=174					
Diag (SD)	£614.93 (465)	£153.46 (365)	£768.39 (535)	£44.93 (210)	£36.32 (173)	£849.64 (620)

	MPS (n=481)					
	Year 1					
	90d	90-365	0-365	Year 2	Year 3	Total†
**LPTL**	n=125					
Diag (SD)	£587.79 (216)	£67.51 (212)	£655.30 (279)	£12.27 (142)	£9.05 (40)	£676.62 (298)
Revas (SD)	0 (0)	£84.56 (666)	£84.56 (666)	£16.92 (133)	0 (0)	£100.90 (899)
Inpatient* (SD)	£49.55 (290)	£44.43 (315)	£93.97 (478)	£0.02 (0.15)	£0.02 (0.20)	£94.02 (478)
Outpatient (SD)	£41.19 (65)	£27.81 (78)	£69.00 (107)	£11.33 (54)	£19.77 (74)	£98.40 (162)
Drug (SD)	£6.16 (14)	£17.39 (29)	£23.55 (40)	£22.84 (39)	£24.49 (46)	£68.48 (110)
Total (SD)	£684.69 (427)	£241.69 (953)	£926.38 (1076)	£61.65 (775)	£50.39 (104)	£1038.42 (1357)
**MPTL**	n=183					
Diag (SD)	£553.57 (247)	£133.16 (339)	£686.73 (400)	£39.75 (230)	£34.75 (179)	£761.23 (500)
Revas (SD)	£86.64 (673)	£144.40 (864)	£231.05 (1084)	£28.88 (391)	£28.88 (391)	£285.91 (1193)
Inpatient* (SD)	£58.47 (341)	£52.70 (273)	£111.78 (430)	£0.01 (0.10)	£0.02 (0.15)	£111.21 (430)
Outpatient (SD)	£40.10 (71)	£47.83 (92)	£87.93 (117)	£10.55 (47)	£7.88 (39)	£105.48 (134)
Drug (SD)	£7.19 (9)	£20.97 (25)	£28.16 (32)	£28.33 (50)	£30.01 (57)	£83.53 (121)
Total (SD)	£745.97 (1026)	£399.07 (1209)	£1145.04 (1571)	£105.23 (596)	£97.09 (481)	£1347.37 (1777)
**HPTL**	n=173					
Diag (SD)	£658.14 (319)	£239.93 (463)	£898.07 (528)	£52.00 (256)	£17.84 (97)	£967.91 (616)

	NICE (n=240)					
	Year 1					
	90d	90-365	0-365	Year 2	Year 3	Total†
**LPTL**	n=61					
Diag (SD)	£286.09 (234)	£134.51 (356)	£420.60 (412)	£10.85 (58)	£14.60 (56)	£446.05 (417)
Revas (SD)	£86.64 (677)	0 (0)	£86.64 (677)	0 (0)	0 (19)	£86.64 (677)
Inpatient* (SD)	£20.31 (159)	£40.61 (222)	£60.92 (270)	£0.03 (0.18)	0 (0)	£60.95 (270)
Outpatient (SD)	£25.32 (52)	£25.32 (66)	£50.65 (79)	£17.30 (65)	£19.42 (58)	£85.49 (128)
Drug (SD)	£5.95 (8)	£17.63 (31)	£23.58 (37)	£26.43 (52)	£31.05 (56)	£78.10 (135)
Total (SD)	£424.31 (863)	£218.08 (518)	£642.38 (991)	£53.14 (126)	£61.72 (114)	£757.24 (1052)
**MPTL**	n=88					
Diag (SD)	£592.87 (227)	£141.72 (392)	£734.59 (446)	£31.08 (205)	£65.58 (244)	£831.24 (614)
Revas (SD)	0 (0)	£300.30 (1468)	£300.30 (1468)	£120.12 (792)	£60.06 (333)	£472.42 (1703)
Inpatient* (SD)	£34.95 (235)	£39.22 (269)	£74.18 (353)	£0.05 (0.34)	£0.06 (0.23)	£74.27 (353)
Outpatient (SD)	£39.50 (63)	£49.74 (94)	£89.23 (113)	£25.16 (98)	£32.18 (116)	£143.59 (231)
Drug (SD)	£8.33 (12)	£31.05 (49)	£39.37 (59)	£47.91 (88)	£53.43 (115)	£135.54 (245)
Total (SD)	£675.65 (380)	£562.02 (1954)	£1237.67 (1996)	£217.78 (976)	£201.61 (635)	£1657.06 (2632)
**HPTL**	n=91					
Diag (SD)	£1000.43 (405)	£86.39 (263)	£1086.82 (291)	£27.33 (107)	£8.12 (48)	£1122.27 (309)

Revas (SD)	£546.74 (1614)	£637.87 (1818)	£1184.61 (2352)	£60.75 (565)	£30.37 (401)	£1271.66 (2464)	£213.85 (1190)	£763.76 (1949)	£977.61 (2210)	£103.87 (1042)	£6.11 (61)	£1083.67 (2491)	£464.63 (1505)	£464.63 (1505)	£929.26 (2023)	0 (0)	0 (0)	£929.26 (2023)
Inpatient* (SD)	£75.27 (336)	£169.58 (766)	£244.85 (821)	£0.04 (0.23)	£0.03 (0.20)	£244.91 (821)	£41.90 (281)	£91.39 (455)	£133.29 (528)	£0.08 (0.33)	£0.05 (0.21)	£133.40 (528)	£40.83 (222)	£68.06 (284)	£108.89 (353)	£0.05 (0.27)	£0.02 (0.15)	£108.96 (353)
Outpatient (SD)	£51.79 (69)	£108.75 (191)	£160.54 (204)	£34.03 (116)	£24.12 (88)	£215.94 (298)	£51.34 (65)	£68.46 (111)	£119.80 (130)	£34.08 (88)	£23.81 (83)	£174.96 (209)	£48.10 (68)	£74.97 (96)	£123.07 (113)	£33.95 (85)	£15.84 (58)	£170.66 (156)
Drug (SD)	£12.11 (13)	£42.34 (52)	£54.50 (62)	£57.21 (68)	£52.84 (57)	£159.11 (168)	£13.74 (24)	£44.98 (73)	£58.73 (89)	£61.41 (107)	£63.81 (112)	£177.62 (281)	£9.06 (7)	£34.61 (33)	£43.67 (38)	£47.63 (42)	£47.48 (42)	£134.02 (104)
Total (SD)	£1300.84 (2085)	£1112.01 (2473)	£2412.89 (3204)	£191.82 (682)	£136.55 (528)	£2741.26 (3392)	£978.98 (1379)	£1208.52 (2449)	£2187.50 (2763)	£244.69 (1253)	£105.38 (318)	£2537.57 (3171)	£1563.06 (1654)	£728.67 (1586)	£2291.72 (2190)	£106.21 (176)	£67.25 (101)	£2465.18 (2218)
**Overall**	**n=481**						**n=481**						**n=240**					
Diag (SD)	£477.03 (381)	£112.37 (318)	£589.40 (469)	£36.99 (180)	£26.37 (177)	£652.76 (556)	£600.07 (272)	£154.50 (369.87)	£754.57 (438.71)	£37.01 (222)	£21.99 (127)	£813.58 (518)	£669.43 (419.17)	£118.91 (338)	£788.34 (465)	£24.52 (143)	£30.83 (10)	£843.69 (535)
Revas (SD)	£296.67 (1218)	£296.67 (1265)	£593.35 (1804)	£42.47 (464)	£24.62 (373)	£660.43 (1908)	£109.88 (828)	£351.61 (1362)	£461.49 (1570)	£50.96 (699)	£12.31 (270)	£524.76 (1765)	£198.19 (1006)	£286.28 (1293)	£484.48 (1603)	£42.55 (465)	£20.56 (318)	£547.59 (1680)
Inpatient* (SD)	£61.13 (331)	£83.70 (534)	£144.84 (673)	£0.05 (0.37)	£0.03 (0.22)	£144.91 (673)	£50.19 (307)	£64.47 (358)	£114.66 (479)	£0.04 (0.21)	£0.03 (0.18)	£114.73 (479)	£33.46 (212)	£50.51 (263)	£83.97 (333)	£0.04 (0.27)	£0.03 (0.16)	£84.04 (333)
Outpatient (SD)	£45.23 (75)	£69.32 (191)	£114.55 (231)	£26.06 (86)	£16.74 (65)	£157.35 (292)	£44.43 (67)	£50.05 (97)	£94.47 (121)	£18.57 (63)	£15.59 (63)	£128.63 (172)	£39.16 (63)	£53.10 (91)	£92.26 (109)	£25.60 (83)	£21.23 (79)	£139.09 (184)
Drug (SD)	£8.00 (11)	£28.38 (54)	£36.39 (62)	£37.30 (79)	£34.62 (64)	£108.32 (195)	£9.28 (17)	£28.68 (50)	£37.95 (62.31)	£37.49 (73)	£38.02 (76)	£113.46 (199)	£8.00 (9)	£28.99 (40)	£36.99 (47)	£40.91 (63)	£42.46 (75)	£120.36 (177)
Total (SD)	£888.07 (1624)	£590.44 (1786)	£1478.52 (2580)	£142.87 (604)	£102.38 (563)	£1723.77 (2828)	£813.85 (1070)	£649.30 (1766)	£1463.15 (2067)	£144.06 (911)	£87.94 (355)	£1695.15 (2375)	£948.24 (1229)	£537.79 (1563)	£1486.03 (1988)	£133.63 (606)	£115.11 (398)	£1734.77 (2259)

*Inpatient care costs from cardiovascular hospitalisations only

† All costs are discounted at 3.5% per annum

LPTL: Low pre-test likelihood; MPTL: Medium pre-test likelihood; HPTL: High pre-test likelihood; CMR: Cardiovascular Magnetic Resonance; MPS: Myocardial Perfusion Scintigraphy; NICE: National Institute for Health and Social Care Excellence Guidance; Diag: Diagnostics; Revas; Revascularisations; SD: Standard deviation

Table S5: Inpatient days and outpatient visits

	CMR (n=481)						MPS (n=481)						NICE (n=240)					
	Year 1						Year 1						Year 1					
	90d	90-365	0-365	Year 2	Year 3	Total	90d	90-365	0-365	Year 2	Year 3	Total	90d	90-365	0-365	Year 2	Year 3	Total
**Low PTL**	**n=128**						**n=125**						**n=61**					
**CV hosp**																		
Patients, N (%)	2 (1.57)	2 (1.57)	3 (2.34)	4 (3.13)	3 (2.34)	6 (4.69)	4 (3.20)	3 (2.40)	6 (4.80)	3 (2.40)	2 (2.40)	10 (8.00)	1 (1.64)	2 (3.28)	3 (4.92)	2 (3.28)	0 (0.00)	4 (6.56)
Total admission days, N (SD)*	4 (0)	5 (2.12)	9 (2.65)	11 (2.87)	6 (1.73)	26 (6.31)	5 (0.5)	4 (0.58)	9 (0.84)	3 (0)	3 (0.71)	15 (0.71)	1 -	2 (0)	3 (0)	2 (0)	0 -	5 (0.5)
**Non CV hosp**																		
Patients, N (%)	2 (1.57)	7 (5.47)	9 (7.03)	9 (7.03)	5 (3.91)	17 (13.3)	3 (2.40)	5 (4.00)	7 (5.60)	8 (6.40)	11 (8.80)	21 (16.80)	4 (6.56)	6 (9.84)	9 (14.75)	4 (6.56)	1 (1.64)	12 (19.67)
Total admission days, N (SD)*	2 (0)	9 (0.76)	11 (0.67)	14 (1.01)	14 (1.64)	39 (2.57)	3 (0)	6 (0.45)	9 (0.76)	11 (1.06)	13 (0.40)	33 (1.03)	4 (0)	8 (0.52)	12 (0.5)	4 (0)	1 -	17 (0.67)
**Outpatients**																		
Patients, N (%)	43 (33.59)	14 (10.94)	52 (40.63)	8 (6.25)	9 (7.03)	55 (42.97)	38 (30.40)	19 (15.20)	49 (39.20)	8 (6.40)	11 (8.80)	55 (44.00)	12 (19.67)	9 (14.75)	20 (32.79)	5 (8.20)	7 (11.48)	26 (42.62)
Total visits, N (SD)*	44 (0.15)	21 (0.76)	65 (0.65)	12 (1.07)	12 (0.71)	89 (1.22)	40 (0.23)	27 (0.84)	67 (0.78)	10 (0.71)	19 (1.01)	96 (1.32)	12 (0)	12 (0.50)	24 (0.41)	8 (0.89)	9 (0.49)	41 (0.99)
**Medium PTL**	**n=179**						**n=183**						**n=88**					
**CV hosp**																		
Patients, N (%)	7 (3.91)	3 (1.68)	9 (5.03)	6 (3.35)	2 (1.11)	16 (8.94)	6 (3.28)	7 (3.83)	13 (7.10)	2 (1.09)	4 (2.19)	15 (8.20)	2 (2.27)	2 (2.27)	4 (4.55)	2 (2.27)	5 (5.68)	8 (9.11)
Total admission days, N (SD)*	8 (0.38)	3 (0)	11 (0.44)	7 (0.41)	2 (0)	20 (0.45)	7 (0.41)	7 (0)	14 (0.28)	2 (0)	4 (0)	20 (0.72)	2 (0)	2 (0)	4 (0)	4 (1.41)	5 (0)	13 (1.41)
**Non CV hosp**																		
Patients, N (%)	6 (3.35)	17 (9.50)	22 (12.29)	18 (10.1)	15 (8.38)	42 (23.46)	2 (1.09)	13 (7.10)	13 (7.10)	17 (9.29)	12 (6.56)	35 (19.13)	0 (0)	4 (4.55)	4 (4.55)	10 (11.36)	9 (10.23)	19 (21.59)
Total admission days, N (SD)*	7 (0.41)	20 (0.39)	27 (0.43)	28 (1.04)	23 (0.84)	78 (1.35)	2 (0)	14 (0.28)	16 (0.60)	17 (0)	19 (1.44)	52 (1.04)	0 -	4 (0)	4 (0)	10 (0)	10 (0.33)	24 (0.45)
**Outpatients**																		

Patients, N (%)	48 (26.82)	50 (27.93)	87 (48.60)	30 (16.76)	17 (9.50)	103 (57.54)	52 (28.42)	51 (27.87)	87 (47.54)	11 (6.01)	8 (4.37)	96 (52.46)	26 (29.54)	25 (28.41)	46 (52.27)	7 (7.95)	9 (10.23)	49 (55.68)
Total visits, N (SD)*	55 (0.87)	91 (3.13)	146 (3.18)	42 (0.67)	21 (0.66)	209 (3.37)	57 (0.45)	68 (0.74)	125 (0.80)	15 (0.67)	11 (0.52)	151 (0.96)	27 (0.20)	34 (0.76)	61 (0.79)	17 (0.79)	21 (1.41)	99 (2.04)
**High PTL**	**n=174**						**n=173**						**n=91**					
**CV hosp**																		
Patients, N (%)	9 (5.17)	11 (6.32)	20 (11.49)	6 (3.45)	4 (2.30)	24 (13.79)	4 (2.31)	9 (5.20)	13 (7.51)	10 (5.78)	8 (4.62)	26 (15.03)	3 (3.30)	5 (5.50)	8 (8.79)	4 (4.40)	2 (2.20)	13 (14.30)
Total admission days, N (SD)*	9 (0)	14 (0.47)	23 (0.37)	7 (0.41)	5 (0.41)	35 (0.78)	5 (0.50)	10 (0.33)	15 (0.38)	13 (0.48)	8 (0)	36 (0.64)	3 (0)	5 (0)	8 (0)	5 (0.5)	2 (0)	15 (0.55)
**Non CV hosp**																		
Patients, N (%)	6 (3.45)	15 (8.62)	20 (11.49)	18 (10.34)	20 (11.49)	46 (26.44)	6 (3.47)	20 (11.56)	22 (12.72)	19 (10.98)	20 (11.56)	46 (26.59)	5 (5.50)	5 (5.50)	9 (9.89)	9 (9.89)	9 (9.89)	22 (24.18)
Total admission days, N (SD)*	8 (0.52)	18 (0.56)	26 (0.66)	25 (0.85)	25 (0.64)	76 (1.34)	6 (0)	24 (0.70)	30 (0.90)	28 (1.12)	25 (0.44)	83 (1.34)	6 (0.45)	6 (0.45)	12 (0.71)	12 (0.71)	10 (0.33)	34 (0.37)
**Outpatients**																		
Patients, N (%)	66 (37.93)	78 (44.83)	117 (67.24)	27 (15.52)	17 (9.77)	126 (72.41)	68 (39.31)	60 (34.68)	104 (60.12)	29 (16.76)	20 (11.56)	115 (66.47)	32 (35.16)	42 (46.15)	62 (68.13)	15 (16.48)	8 (8.79)	66 (72.53)
Total visits, N (SD)*	70 (0.24)	147 (1.73)	217 (1.62)	46 (1.68)	32 (1.27)	295 (2.49)	69 (0.12)	92 (0.77)	161 (0.86)	44 (0.74)	30 (1.10)	235 (1.64)	34 (0.25)	53 (0.59)	87 (0.71)	24 (0.74)	11 (0.74)	122 (1.08)
**Overall**	**n=481**						**n=481**						**n=240**					
**CV hosp**																		
Patients, N (%)	18 (3.74)	16 (3.33)	32 (6.65)	16 (3.33)	9 (1.87)	46 (9.56)	14 (2.91)	19 (3.95)	32 (6.65)	15 (3.11)	14 (2.91)	51 (10.60)	6 (2.50)	9 (3.75)	15 (6.25)	8 (3.33)	7 (2.92)	25 (10.42)
Total admission days, N (SD)*	21 (0.38)	22 (0.80)	43 (0.94)	25 (1.50)	13 (1.01)	81 (0.67)	17 (0.43)	21 (0.32)	38 (0.47)	18 (0.41)	15 (0.41)	71 (0.27)	6 (0)	9 (0)	15 (0)	11 (0.74)	7 (0)	33 (0.90)
**Non CV hosp**																		
Patients, N (%)	14 (2.91)	39 (8.10)	51 (10.60)	45 (9.36)	40 (8.32)	105 (21.83)	11 (2.29)	38 (7.90)	42 (8.73)	44 (9.15)	43 (8.94)	102 (21.21)	9 (3.75)	15 (6.25)	22 (9.16)	23 (9.58)	19 (7.92)	53 (22.08)
Total admission days, N (SD)*	17 (0.43)	47 (0.52)	64 (0.56)	67 (0.94)	62 (0.99)	193 (1.60)	11 (0)	44 (0.55)	55 (0.78)	56 (0.87)	57 (0.83)	168 (1.18)	10 (0.33)	18 (0.41)	28 (0.55)	26 (0.46)	21 (0.32)	75 (0.63)
**Outpatients**																		

Patients, N (%)	157 (32.64)	142 (29.52)	256 (53.22)	65 (13.50)	43 (8.94)	287 (59.67)	158 (32.85)	130 (27.03)	240 (49.90)	48 (9.98)	39 (8.11)	266 (55.30)	70 (29.17)	76 (31.67)	128 (53.33)	27 (11.25)	24 (10.00)	141 (58.75)
Total visits, N (SD)*	169 (0.51)	259 (2.26)	428 (2.18)	100 (1.23)	65 (0.98)	593 (2.67)	166 (0.29)	187 (0.77)	353 (0.82)	69 (0.71)	60 (0.97)	482 (1.37)	73 (0.20)	99 (0.63)	172 (0.70)	49 (1.00)	41 (1.08)	262 (1.47)

*SD of total visits conditional on having attended a given visit (outpatient or hospital visit >0)

PTL: Pre-test likelihood; CMR: Cardiovascular Magnetic Resonance; MPS: Myocardial Perfusion Scintigraphy; NICE: National Institute for Health and Social Care Excellence Guidance; CV: Cardiovascular; Hosp: Hospitalisation; SD: Standard deviation

Table S6: Mean EQ-5D scores over time

CMR (n=481)

HRQoL	Baseline	Month 6	Month 12	Month 24	Month 36
**LPTL**	**n=174**				
EQ5D-3L (SD)	0.77233 (0.24224)	0.80528 (0.26300)	0.80313 (0.31289)	0.81139 (0.29942)	0.79616 (0.27151)
EQ5D-5L (SD)	0.86384 (0.15977)	0.86986 (0.21015)	0.86863 (0.22650)	0.86689 (0.21203)	0.86136 (0.19420)
**MPTL**	**n=179**				
EQ5D-3L (SD)	0.76384 (0.21449)	0.77651 (0.27611)	0.76296 (0.38639)	0.78243 (0.29070)	0.75450 (0.33974)
EQ5D-5L (SD)	0.86384 (0.15977)	0.86986 (0.21015)	0.86863 (0.22650)	0.86689 (0.21203)	0.86136 (0.19420)
**HPTL**	**n=174**				
EQ5D-3L (SD)	0.72799 (0.24663)	0.73020 (0.30541)	0.76452 (0.30269)	0.74966 (0.35411)	0.74820 (0.31378)
EQ5D-5L (SD)	0.83033 (0.16640)	0.80813 (0.23432)	0.82432 (0.24231)	0.82467 (0.25232)	0.82424 (0.25200)
**Overall**	**n=481**				
EQ5D-3L (SD)	0.75313 (0.23457)	0.76741 (0.27520)	0.77421 (0.37154)	0.77828 (0.31824)	0.76331 (0.33970)
EQ5D-5L (SD)	0.84948 (0.16585)	0.84115 (0.21471)	0.84108 (0.25888)	0.84223 (0.23487)	0.83598 (0.24431)

MPS (n=481)

HRQoL	Baseline	Month 6	Month 12	Month 24	Month 36
**LPTL**	**n=173**				
EQ5D-3L (SD)	0.75902 (0.23003)	0.76774 (0.29015)	0.78106 (0.26446)	0.77114 (0.29219)	0.75798 (0.26972)
EQ5D-5L (SD)	0.84975 (0.17356)	0.84739 (0.19681)	0.85302 (0.20995)	0.85361 (0.19385)	0.83702 (0.20665)
**MPTL**	**n=183**				
EQ5D-3L (SD)	0.72962 (0.24899)	0.75952 (0.30298)	0.75919 (0.31517)	0.76622 (0.36804)	0.74980 (0.32653)
EQ5D-5L (SD)	0.84975 (0.17356)	0.84739 (0.19681)	0.85302 (0.20995)	0.85361 (0.19385)	0.83702 (0.20665)
**HPTL**	**n=173**				
EQ5D-3L (SD)	0.73447 (0.22163)	0.74562 (0.27108)	0.72780 (0.30824)	0.73788 (0.30931)	0.71283 (0.30782)
EQ5D-5L (SD)	0.82764 (0.16730)	0.83726 (0.18628)	0.81612 (0.21959)	0.81628 (0.22652)	0.79672 (0.23957)
**Overall**	**n=481**				
EQ5D-3L (SD)	0.73900 (0.23402)	0.75666 (0.29467)	0.75358 (0.31099)	0.75731 (0.34803)	0.73863 (0.30325)
EQ5D-5L (SD)	0.83791 (0.17641)	0.83903 (0.22834)	0.83266 (0.25239)	0.83300 (0.23313)	0.81892 (0.23098)

NICE (n=240)

HRQoL	Baseline	Month 6	Month 12	Month 24	Month 36
**LPTL**	**n=91**				
EQ5D-3L (SD)	0.71049 (0.26377)	0.73620 (0.28165)	0.72778 (0.32542)	0.74110 (0.31676)	0.72520 (0.36215)
EQ5D-5L (SD)	0.81202 (0.19411)	0.80581 (0.24266)	0.81294 (0.25672)	0.83026 (0.22739)	0.80585 (0.27487)
**MPTL**	**n=88**				
EQ5D-3L (SD)	0.73072 (0.20962)	0.69722 (0.25118)	0.68143 (0.29992)	0.68081 (0.27797)	0.69832 (0.28889)
EQ5D-5L (SD)	0.81202 (0.19411)	0.80581 (0.24266)	0.81294 (0.25672)	0.83026 (0.22739)	0.80585 (0.27487)
**HPTL**	**n=91**				
EQ5D-3L (SD)	0.71039 (0.28053)	0.75393 (0.26466)	0.73691 (0.32133)	0.71203 (0.37407	0.72097 (0.32049)
EQ5D-5L (SD)	0.81809 (0.20994)	0.82740 (0.20996)	0.80842 (0.26446)	0.80649 (0.26858)	0.78670 (0.26084)
**Overall**	**n=240**				
EQ5D-3L (SD)	0.71787 (0.26595)	0.72863 (0.28293)	0.71425 (0.33567)	0.70797 (0.35003)	0.71374 (0.29864)
EQ5D-5L (SD)	0.82088 (0.19567)	0.81141 (0.22047)	0.80316 (0.25041)	0.80553 (0.24005)	0.79340 (0.23533)

LPTL: Low pre-test likelihood; MPTL: Medium pre-test likelihood; HPTL: High pre-test likelihood; MPS: Myocardial Perfusion Scintigraphy; CMR: Cardiovascular Magnetic Resonance; NICE: National Institute for Health and Social Care Excellence Guidance; SD: Standard deviation

Table S7: Cost-effectiveness results – not controlling for differential revascularisation rates across arms

	Mean cost per patient (95% CI) [P(most costly)]	Mean QALYs per patient (95% CI) [P(most effective)]	ICER	Incremental net health benefit per patient* (95% CI) [Probability of being cost-effective] k=£15,000	k=£20,000	k=£30,000
**Low PTL**						
NICE	£739.72<br>(481.39, 1051.59)<br>[0.052]	2.20158<br>(2.05259, 2.35016)<br>[0.156]		-<br>-<br>[0.191]	-<br>-<br>[0.184]	-<br>-<br>[0.176]
CMR	£890.25<br>(683.91, 1161.4)<br>[0.156]	2.27686<br>(2.1842, 2.37961)<br>[0.640]	£1,999.70	0.065<br>(-0.113, 0.244)<br>[0.642]	0.068<br>(-0.110, 0.246)<br>[0.647]	0.070<br>(-0.103, 0.247)<br>[0.648]
MPS	£1,066.30<br>(797.17, 1403.31)<br>[0.792]	2.22813<br>(2.1358, 2.31654)<br>[0.204]	Dominated	0.005<br>(-0.167, 0.184)<br>[0.167]	0.010<br>(-0.161, 0.187)<br>[0.169]	0.016<br>(-0.159, 0.191)<br>[0.176]
**Medium PTL**						
CMR	£1,314.13<br>(1035.03, 1660.59)<br>[0.109]	2.21530<br>(2.10543, 2.31045)<br>[0.355]		0.185<br>(0.016, 0.349)<br>[0.372]	0.179<br>(0.019, 0.34)<br>[0.364]	0.174<br>(0.013, 0.334)<br>[0.360]
MPS	£1,322.85<br>(1033.82, 1656.76)<br>[0.133]	2.23666<br>(2.14194, 2.33298)<br>[0.639]	£408.36	0.205<br>(0.055, 0.358)<br>[0.623]	0.200<br>(0.051, 0.351)<br>[0.631]	0.195<br>(0.048, 0.345)<br>[0.635]
NICE	£1,619.41<br>(1157.65, 2229.18)<br>[0.758]	2.05109<br>(1.94005, 2.15466)<br>[0.006]	Dominated	-<br>-<br>[0.005]	-<br>-<br>[0.005]	-<br>-<br>[0.005]
**High PTL**						
NICE	£2,484.19<br>(1831.71, 3357.69)<br>[0.243]	2.17658<br>(2.06393, 2.29351)<br>[0.388]		-<br>-<br>[0.437]	-<br>-<br>[0.435]	-<br>-<br>[0.418]
MPS	£2,504.96<br>(1995.02, 3112.89<br>[0.208]	2.14166<br>(2.05664, 2.22632)<br>[0.082]	Dominated	-0.036<br>(-0.202, 0.120)<br>[0.129]	-0.036<br>(-0.197, 0.114)<br>[0.116]	-0.036<br>(-0.192, 0.109)<br>[0.107]
CMR	£2,728.52<br>(2143.58, 3362.41)<br>[0.549]	2.19613<br>(2.10918, 2.28684)<br>[0.530]	£12,495.02	0.003<br>(-0.155, 0.171)<br>[0.434]	0.007<br>(-0.147, 0.17)<br>[0.449]	0.011<br>(-0.136, 0.162)<br>[0.475]
**Overall**						
MPS	£1,686.59<br>(1445.14, 1956.28)<br>[0.262]	2.19981<br>(2.14269, 2.25958)<br>[0.241]		0.065<br>(-0.024, 0.157)<br>[0.040]	0.065<br>(-0.023, 0.155)<br>[0.042]	0.064<br>(-0.022, 0.153)<br>[0.243]
NICE	£1,704.73<br>(1380.66, 2074.36<br>[0.36]	2.13613<br>(2.07349, 2.20272)<br>[0.014]	Dominated	-<br>-<br>[0.029]	-<br>-<br>[0.032]	-<br>-<br>[0.016]
CMR	£1,718.79<br>(1473.43, 1991.9)<br>[0.378]	2.22440<br>(2.16292, 2.28355)<br>[0.745]	£1,309.99	0.087<br>(-0.005, 0.182)<br>[0.931]	0.088<br>(-0.004, 0.18)<br>[0.926]	0.088<br>(-0.003, 0.179)<br>[0.741]

PTL: Pre-test likelihood; QALY: Quality-adjusted life year; CMR: Cardiovascular Magnetic Resonance; MPS: Myocardial Perfusion Scintigraphy; NICE: National Institute for Health and Social Care Excellence Guidance; ICER: Incremental Cost-Effectiveness Ratio; k: Cost-effectiveness threshold; CI: Credible intervals; P(most costly): Probability of a strategy being the most costly

alternative; P(most effective): Probability of a strategy being the most effective alternative (i.e. highest QALY gain); * All Incremental Net Health Benefits are estimated compared to NICE guided care.

Table S8: Cost-effectiveness results – NHS tariffs

	Mean cost per patient (95% CI) [P(most costly)]	Mean QALYs per patient (95% CI) [P(most effective)]	ICER	Incremental net health benefit per patient* (95% CI) [Probability of being cost-effective] k=£15,000	k=£20,000	k=£30,000
**Low PTL**						
NICE	£727.67 (461.82, 979.66) [0.132]	2.20095 (2.05635, 2.36087) [0.13]		- - [0.158]	- - [0.155]	- - [0.15]
MPS	£791.10 (598.28, 988.41) [0.249]	2.22261 (2.13167, 2.3122) [0.151]	Extendedly Dominated	0.017 (-0.157, 0.187) [0.163]	0.018 (-0.155, 0.186) [0.156]	0.020 (-0.15, 0.185) [0.153]
CMR	£875.51 (627.83, 1124.8) [0.619]	2.28702 (2.19106, 2.38562) [0.719]	£1,717.81	0.076 (-0.104, 0.251) [0.679]	0.079 (-0.1, 0.257) [0.689]	0.081 (-0.095, 0.258) [0.697]
**Medium PTL**						
MPS	£1,135.14 (856.32, 1400.34) [0.092]	2.17327 (2.0717, 2.26984) [0.353]		0.190 (0.018, 0.354) [0.422]	0.187 (0.019, 0.35) [0.396]	0.184 (0.016, 0.353) [0.381]
NICE	£1,309.26 (908.34, 1730.03) [0.458]	1.99474 (1.85789, 2.12644) [0.005]	Dominated	- - [0.003]	- - [0.005]	- - [0.004]
CMR	£1,311.50 (1027.85, 1585.37) [0.45]	2.19738 (2.09621, 2.29582) [0.642]	£7,317.38	0.202 (0.029, 0.378) [0.575]	0.203 (0.029, 0.380) [0.599]	0.203 (0.028, 0.379) [0.615]
**High PTL**						
MPS	£2,347.23 (1834.71, 2833.75) [0.135]	1.97525 (1.76637, 2.18188) [0.040]		-0.057 (-0.344, 0.231) [0.059]	-0.061 (-0.346, 0.226) [0.050]	-0.066 (-0.344, 0.219) [0.046]
CMR	£2,532.14 (2104.26, 2988.22 [0.351]	2.15591 (2.06893, 2.24084) [0.747]	£1,023.51	0.111 (-0.176, 0.413) [0.728]	0.110 (-0.185, 0.41) [0.738]	0.109 (-0.183, 0.407) [0.745]
NICE	£2,604.59 (2009.1, 3273.76) [0.514]	2.04944 (1.78434, 2.3057) [0.213]	Dominated	- - [0.213]	- - [0.212]	- - [0.209]
**Overall**						
MPS	£1,486.94 (1268.57, 1691.46) [0.068]	2.11400 (2.01256, 2.2169) [0.048]		0.055 (-0.087, 0.2) [0.081]	0.053 (-0.089, 0.194) [0.072]	0.050 (-0.093, 0.193) [0.065]
NICE	£1,629.34 (1351.49, 1912.53 [0.451]	2.06854 (1.92352, 2.20276) [0.035]	Dominated	- - [0.034]	- - [0.033]	- - [0.035]
CMR	£1,642.40 (1438.95, 1843.74) [0.481]	2.20568 (2.14564, 2.26468) [0.917]	£1,695.61	0.136 (-0.014, 0.3) [0.885]	0.136 (-0.015, 0.301) [0.895]	0.137 (-0.015, 0.298) [0.900]

PTL: Pre-test likelihood; QALY: Quality-adjusted life year; CMR: Cardiovascular Magnetic Resonance; MPS: Myocardial Perfusion Scintigraphy; NICE: National Institute for Health and Social Care Excellence Guidance; ICER: Incremental Cost-Effectiveness Ratio; k: Cost-effectiveness threshold; CI: Credible intervals; P(most costly): Probability of a strategy being the most costly alternative; P(most effective): Probability of a strategy being the most effective alternative (i.e. highest QALY gain); * All Incremental Net Health Benefits are estimated compared to NICE guided care.

Table S9: Cost-effectiveness results – using only diagnostic costs

	Mean cost per patient (95% CI) [P(most costly)]	Mean QALYs per patient (95% CI) [P(most effective)]	ICER	Incremental net health benefit per patient* (95% CI) [Probability of being cost-effective] k=£15,000	k=£20,000	k=£30,000
**Low PTL**						
NICE	£430.21<br>(361.9, 506.77)<br>[0]	2.20095<br>(2.05635, 2.36087)<br>[0.13]		-<br>-<br>[0.152]	-<br>-<br>[0.144]	-<br>-<br>[0.139]
CMR	£458.95<br>(407.53, 510.5)<br>[0]	2.28702<br>(2.19106, 2.38562)<br>[0.719]	£333.92	0.084<br>(-0.091, 0.254)<br>[0.751]	0.085<br>(-0.089, 0.258)<br>[0.745]	0.085<br>(-0.089, 0.259)<br>[0.738]
MPS	£695.64<br>(618.07, 772.56)<br>[1]	2.22261<br>(2.13167, 2.3122)<br>[0.151]	Dominated	0.004<br>(-0.166, 0.165)<br>[0.097]	0.008<br>(-0.162, 0.168)<br>[0.111]	0.013<br>(-0.158, 0.173)<br>[0.123]
**Medium PTL**						
CMR	£581.88<br>(527.79, 637.41)<br>[0]	2.19738<br>(2.09621, 2.29582)<br>[0.642]		0.219<br>(0.044, 0.393)<br>[0.728]	0.215<br>(0.040, 0.388)<br>[0.715]	0.211<br>(0.037, 0.384)<br>[0.69]
MPS	£768.27<br>(698.86, 836.77)<br>[0.207]	2.17327<br>(2.0717, 2.26984)<br>[0.353]	Dominated	0.182<br>(0.017, 0.351)<br>[0.27]	0.181<br>(0.018, 0.352)<br>[0.281]	0.18<br>(0.018, 0.352)<br>[0.305]
NICE	£821.61<br>(719.68, 929.05)<br>[0.793]	1.99474<br>(1.85789, 2.12644)<br>[0.005]	Dominated	-<br>-<br>[0.002]	-<br>-<br>[0.004]	-<br>-<br>[0.005]
**High PTL**						
CMR	£827.45<br>(747.98, 905.72)<br>[0]	2.15591<br>(2.06893, 2.24084)<br>[0.747]		0.126<br>(-0.172, 0.417)<br>[0.778]	0.121<br>(-0.177, 0.413)<br>[0.769]	0.116<br>(-0.18, 0.408)<br>[0.757]
MPS	£988.42<br>(885.36, 1086.84<br>[0.067]	1.97525<br>(1.76637, 2.18188)<br>[0.04]	Dominated	-0.065<br>(-0.343, 0.222)<br>[0.034]	-0.067<br>(-0.346, 0.22)<br>[0.036]	-0.07<br>(-0.348, 0.215)<br>[0.040]
NICE	£1,123.81<br>(983.39, 1262.74)<br>[0.933]	2.04944<br>(1.78434, 2.3057)<br>[0.213]	Dominated	-<br>-<br>[0.188]	-<br>-<br>[0.195]	-<br>-<br>[0.203]
**Overall**						
CMR	£639.25<br>(602.57, 677.34)<br>[0]	2.20568<br>(2.14564, 2.26468)<br>[0.917]		0.150<br>(-0.004, 0.31)<br>[0.946]	0.147<br>(-0.008, 0.307)<br>[0.941]	0.143<br>(-0.011, 0.305)<br>[0.937]
NICE	£829.48<br>(761.58, 893.69<br>[0.488]	2.06854<br>(1.92352, 2.20276)<br>[0.035]	Dominated	-<br>-<br>[0.026]	-<br>-<br>[0.028]	-<br>-<br>[0.030]
MPS	£829.52<br>(781.08, 877.02)<br>[0.512]	2.11400<br>(2.01256, 2.2169)<br>[0.048]	Dominated	0.045<br>(-0.098, 0.19)<br>[0.028]	0.045<br>(-0.098, 0.189)<br>[0.031]	0.045<br>(-0.098, 0.189)<br>[0.033]

PTL: Pre-test likelihood; QALY: Quality-adjusted life year; CMR: Cardiovascular Magnetic Resonance; MPS: Myocardial Perfusion Scintigraphy; NICE: National Institute for Health and Social Care Excellence Guidance; ICER: Incremental Cost-Effectiveness Ratio; k: Cost-effectiveness threshold; CI: Credible intervals; P(most costly): Probability of a strategy being the most costly alternative; P(most effective): Probability of a strategy being the most effective alternative (i.e. highest QALY gain); * All Incremental Net Health Benefits are estimated compared to NICE guided care.

Table S10: Cost-effectiveness results – Revised MPS unit costs

	Mean cost per patient (95% CI) [P(most costly)]	Mean QALYs per patient (95% CI) [P(most effective)]	ICER	Incremental net health benefit per patient* (95% CI) [Probability of being cost-effective] k=£15,000	k=£20,000	k=£30,000
**Low PTL**						
NICE	£775.50 (505.36, 1045.58) [0.255]	2.20095 (2.05635, 2.36087) [0.130]		- - [0.148]	- - [0.145]	- - [0.143]
MPS	£806.95 (594.12, 1025.03) [0.284]	2.22261 (2.13167, 2.3122) [0.151]	Extendedly dominated	0.020 (-0.158, 0.18) [0.162]	0.020 (-0.154, 0.178) [0.162]	0.021 (-0.152, 0.179) [0.158]
CMR	£844.72 (582.08, 1103.46) [0.461]	2.28702 (2.19106, 2.38562) [0.719]	£804.33	0.081 (-0.095, 0.25) [0.690]	0.083 (-0.092, 0.264) [0.693]	0.084 (-0.094, 0.264) [0.699]
**Medium PTL**						
MPS	£1,143.37 (840.02, 1454.66) [0.114]	2.17327 (2.0717, 2.26984) [0.353]		0.190 (0.018, 0.359) [0.432]	0.187 (0.017, 0.356) [0.407]	0.184 (0.015, 0.349) [0.388]
CMR	£1,308.54 (1028.11, 1608.83) [0.41]	2.19738 (2.09621, 2.29582) [0.642]	£6,853.24	0.203 (0.026, 0.387) [0.565]	0.203 (0.027, 0.387) [0.590]	0.203 (0.028, 0.385) [0.608]
NICE	£1,313.72 (855.21, 1763.46) [0.476]	1.99474 (1.85789, 2.12644) [0.005]	Dominated	- - [0.003]	- - [0.003]	- - [0.004]
**High PTL**						
MPS	£2,398.76 (1852.88, 2939.04) [0.187]	1.97525 (1.76637, 2.18188) [0.040]		-0.059 (-0.337, 0.22) [0.043]	-0.063 (-0.339, 0.212) [0.042]	-0.067 (-0.343, 0.212) [0.041]
CMR	£2,511.51 (2038.15, 2962.36 [0.309]	2.15591 (2.06893, 2.24084) [0.747]	£624.07	0.114 (-0.181, 0.414) [0.750]	0.112 (-0.189, 0.414) [0.746]	0.110 (-0.187, 0.406) [0.749]
NICE	£2,621.23 (1963.65, 3255.29) [0.504]	2.04944 (1.78434, 2.3057) [0.213]	Dominated	- - [0.207]	- - [0.212]	- - [0.210]
**Overall**						
MPS	£1,512.94 (1272.39, 1743.27) [0.120]	2.11400 (2.01256, 2.2169) [0.048]		0.055 (-0.088, 0.199) [0.070]	0.052 (-0.091, 0.194) [0.068]	0.050 (-0.093, 0.193) [0.061]
CMR	£1,625.73 (1399.85, 1855.58 [0.364]	2.20568 (2.14564, 2.26468) [0.917]	£1,230.20	0.139 (-0.012, 0.301) [0.900]	0.138 (-0.014, 0.301) [0.901]	0.138 (-0.015, 0.3) [0.907]
NICE	£1,649.57 (1339.94, 1942.59) [0.516]	2.06854 (1.92352, 2.20276) [0.035]	Dominated	- - [0.030]	- - [0.031]	- - [0.032]

PTL: Pre-test likelihood; QALY: Quality-adjusted life year; CMR: Cardiovascular Magnetic Resonance; MPS: Myocardial Perfusion Scintigraphy; NICE: National Institute for Health and Social Care Excellence Guidance; ICER: Incremental Cost-Effectiveness Ratio; k: Cost-effectiveness threshold; CI: Credible intervals; P(most costly): Probability of a strategy being the most costly alternative; P(most effective): Probability of a strategy being the most effective alternative (i.e. highest QALY gain); * All Incremental Net Health Benefits are estimated compared to NICE guided care.

Table S11: Cost-effectiveness results – EQ-5D-5L

	Mean cost per patient (95% CI) [P(most costly)]	Mean QALYs per patient (95% CI) [P(most effective)]	ICER	Incremental net health benefit per patient* (95% CI) k=£15,000 [Probability of being cost-effective]	k=£20,000	k=£30,000
**Low PTL**						
NICE	£787.92 (531.64, 1046.85) [0.047]	2.43234 (2.30617, 2.55652) [0.196]		- - [0.237]	- - [0.226]	- - [0.215]
CMR	£846.00 (597.5, 1098.28) [0.083]	2.47754 (2.41103, 2.54612) [0.518]	£1,285.02	0.041 (-0.100, 0.174) [0.554]	0.042 (-0.095, 0.176) [0.541]	0.043 (-0.092, 0.174) [0.534]
MPS	£1,061.94 (835.89, 1293.69) [0.87]	2.45839 (2.39336, 2.52784) [0.286]	Dominated	0.008 (-0.132, 0.153) [0.209]	0.012 (-0.127, 0.157) [0.233]	0.017 (-0.121, 0.159) [0.251]
**Medium PTL**						
CMR	£1,301.97 (1056.23, 1560.61) [0.074]	2.41772 (2.34753, 2.4927) [0.577]		0.132 (-0.001, 0.268) [0.620]	0.128 (-0.005, 0.262) [0.607]	0.123 (-0.009, 0.255) [0.597]
MPS	£1,391.20 (1105.68, 1684.8) [0.211]	2.40701 (2.32757, 2.48568) [0.407]	Dominated	0.115 (-0.007, 0.238) [0.368]	0.112 (-0.012, 0.234) [0.381]	0.110 (-0.014, 0.230) [0.391]
NICE	£1,565.28 (1114.65, 1991.53) [0.715]	2.30330 (2.20057, 2.40606) [0.016]	Dominated	- - [0.012]	- - [0.012]	- - [0.012]
**High PTL**						
CMR	£2,514.86 (2058.12, 2936.73) [0.184]	2.36506 (2.30222, 2.42664) [0.620]		0.112 (-0.117, 0.326) [0.638]	0.110 (-0.116, 0.320) [0.635]	0.108 (-0.113, 0.316) [0.624]
NICE	£2,638.37 (2048.84, 3213.82 [0.400]	2.26125 (2.07755, 2.45721) [0.081]	Dominated	- - [0.080]	- - [0.079]	- - [0.080]
MPS	£2,660.93 (2154.92, 3139.48) [0.416]	2.31846 (2.1235, 2.5106) [0.299]	Dominated	0.056 (-0.142, 0.257) [0.282]	0.056 (-0.139, 0.258) [0.286]	0.056 (-0.137, 0.255) [0.296]
**Overall**						
CMR	£1,624.82 (1431.4, 1824.44) [0.073]	2.41415 (2.37377, 2.45526) [0.665]		0.101 (-0.015, 0.216) [0.721]	0.099 (-0.016, 0.213) [0.708]	0.097 (-0.018, 0.210) [0.695]
NICE	£1,753.24 (1473.15, 2031.75 [0.440]	2.32169 (2.21917, 2.42485) [0.017]	Dominated	- - [0.017]	- - [0.018]	- - [0.018]
MPS	£1,767.87 (1571.78, 1989.15) [0.487]	2.38816 (2.29151, 2.48441) [0.318]	Dominated	0.065 (-0.043, 0.173) [0.262]	0.066 (-0.044, 0.173) [0.274]	0.066 (-0.044, 0.172) [0.287]

PTL: Pre-test likelihood; QALY: Quality-adjusted life year; CMR: Cardiovascular Magnetic Resonance; MPS: Myocardial Perfusion Scintigraphy; NICE: National Institute for Health and Social Care Excellence Guidance; ICER: Incremental Cost-Effectiveness Ratio; k: Cost-effectiveness threshold; CI: Credible intervals; P(most costly): Probability of a strategy being the most costly alternative; P(most effective): Probability of a strategy being the most effective alternative (i.e. highest QALY gain); * All Incremental Net Health Benefits are estimated compared to NICE guided care.

Table S12: Cost-effectiveness results – Pooled functional imaging arm (PFIA)

	Mean cost per patient (95% CI) [P(most costly)]	Mean QALYs per patient (95% CI) [P(most effective)]	ICER	Incremental net health benefit per patient* (95% CI) [Probability of being cost-effective] k=£15,000	k=£20,000	k=£30,000
**Low PTL**						
NICE	£794.34 (552.62, 1044.48) [0.136]	2.15534 (2.01022, 2.30508) [0.099]		- - [0.135]	- - [0.126]	- - [0.116]
PFIA	£963.27 (808.17, 1141.51) [0.864]	2.25924 (2.19489, 2.32185) [0.901]	£1,625.92	0.093 (-0.071, 0.248) [0.865]	0.095 (-0.066, 0.25) [0.874]	0.098 (-0.063, 0.256) [0.884]
**Medium PTL**						
PFIA	£1,344.22 (1148.78, 1527.8) [0.176]	2.20270 (2.12959, 2.28331) [0.989]		0.187 (0.036, 0.330) [0.995]	0.184 (0.036, 0.322) [0.994]	0.180 (0.034, 0.321) [0.992]
NICE	£1,563.73 (1167.66, 1989.85) [0.824]	2.02996 (1.92345, 2.13508) [0.011]	Dominated	- - [0.005]	- - [0.006]	- - [0.008]
**High PTL**						
PFIA	£2,582.19 (2254.13, 2910.9) [0.432]	2.13792 (2.08412, 2.19966) [0.486]		-0.003 (-0.149, 0.131) [0.504]	-0.004 (-0.144, 0.125) [0.500]	-0.004 (-0.143, 0.119) [0.491]
NICE	£2,620.38 (2008.4, 3169.52) [0.568]	2.14353 (2.03009, 2.2639) [0.514]	£6,807.25	- - [0.496]	- - [0.500]	- - [0.509]
**Overall**						
PFIA	£1,695.81 (1548.77, 1823.17) [0.369]	2.19386 (2.15065, 2.23733) [0.981]		0.093 (0.004, 0.176) [0.98]	0.092 (0.004, 0.173) [0.98]	0.091 (0.005, 0.170) [0.979]
NICE	£1,747.78 (1486.11, 2019.52) [0.631]	2.10410 (2.03778, 2.17674) [0.019]	Dominated	- - [0.020]	- - [0.020]	- - [0.021]

PTL: Pre-test likelihood; QALY: Quality-adjusted life year; PFIA: Pooled Functional Imaging Arm; NICE: National Institute for Health and Social Care Excellence Guidance; ICER: Incremental Cost-Effectiveness Ratio; k: Cost-effectiveness threshold; CI: Credible intervals; P(most costly): Probability of a strategy being the most costly alternative; P(most effective): Probability of a strategy being the most effective alternative (i.e. highest QALY gain); * All Incremental Net Health Benefits are estimated compared to NICE guided care.

Table S13: Cost of CMR at which it is no longer cost-effective

	k=£15,000	k=£20,000	k=£30,000
	Cost of CMR at which it is no longer cost-effective	Cost of CMR at which it is no longer cost-effective	Cost of CMR at which it is no longer cost-effective
	[Increment from base case]	[Increment from base case]	[Increment from base case]
Low PTL	£1,698	£2,053	£2,764
	[£1304]	[£1660]	[£2370]
Med PTL	£938	£1,084	£1,375
	[£545]	[£690]	[£981]
High PTL	£2,331	£2,931	£4,129
	[£1937]	[£2537]	[£3736]
Overall	£2,112	£2,631	£3,668
	[£1718]	[£2237]	[£3275]

Figure S1: Cost-effectiveness acceptability curves (base case analysis)

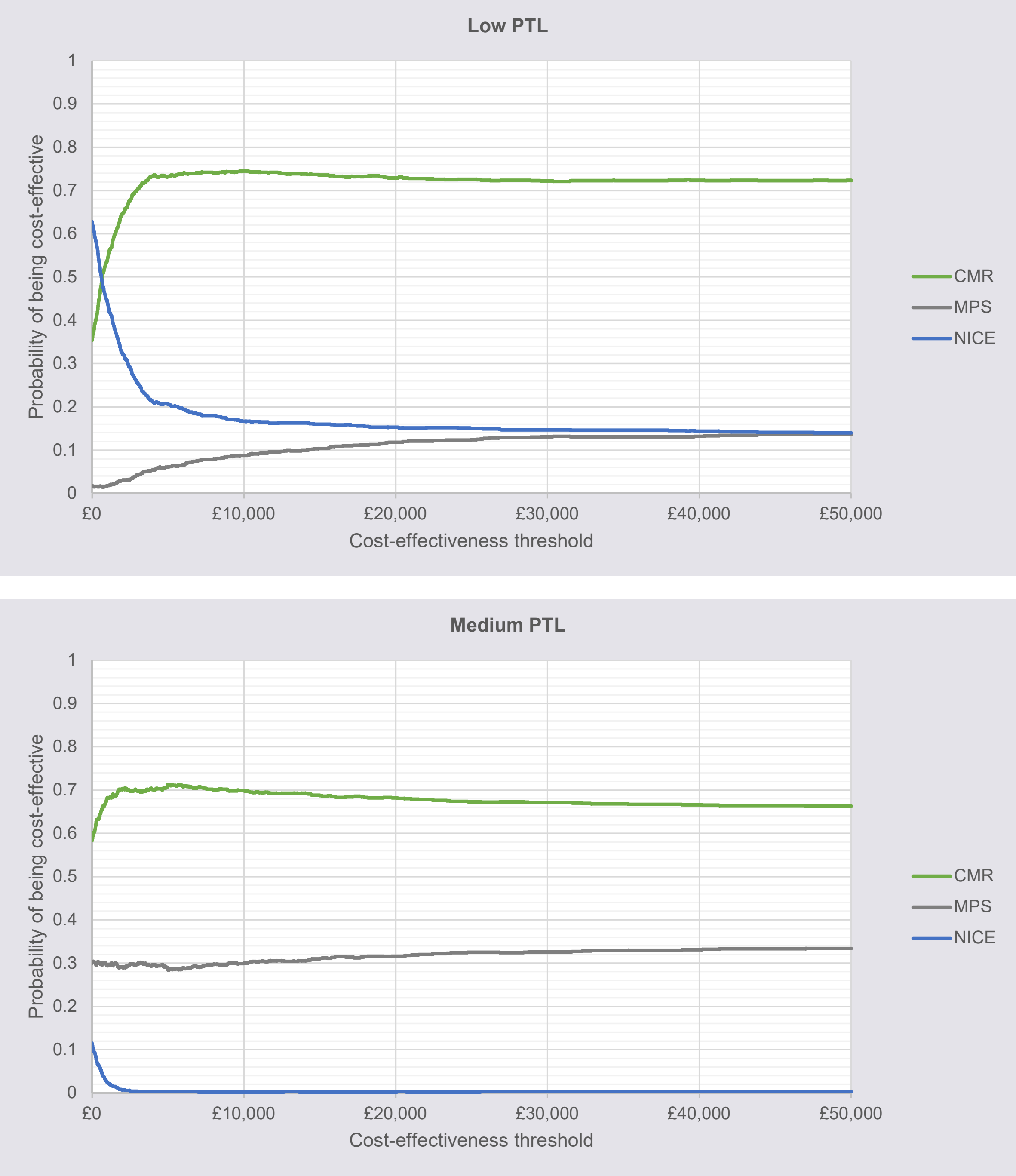

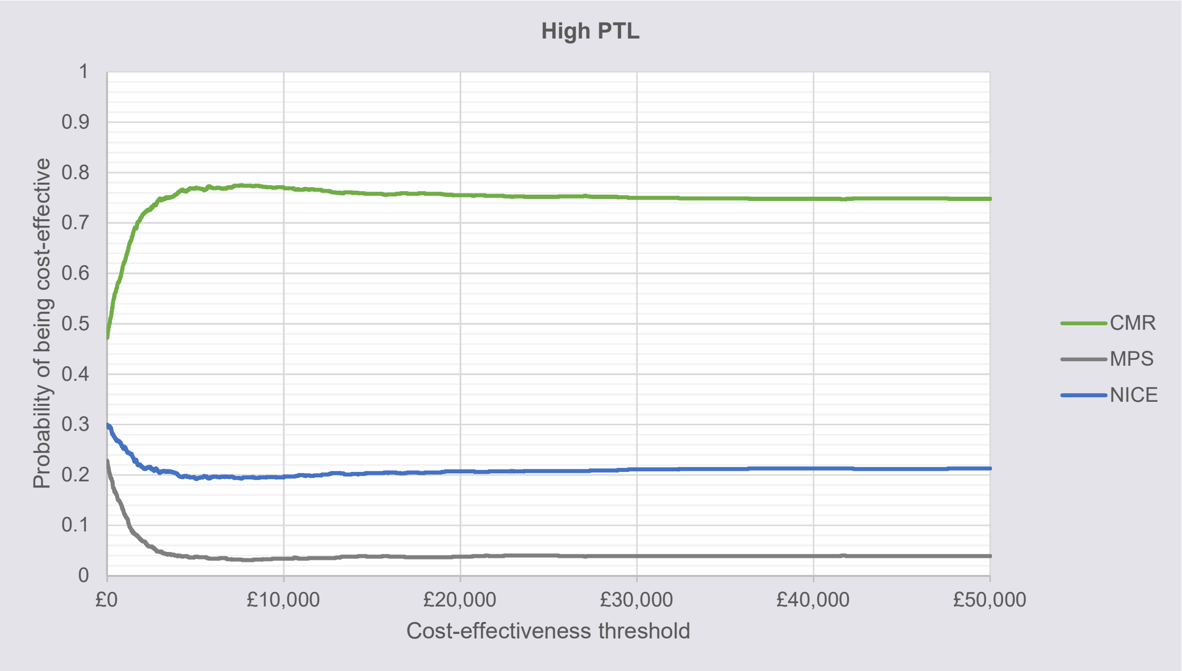

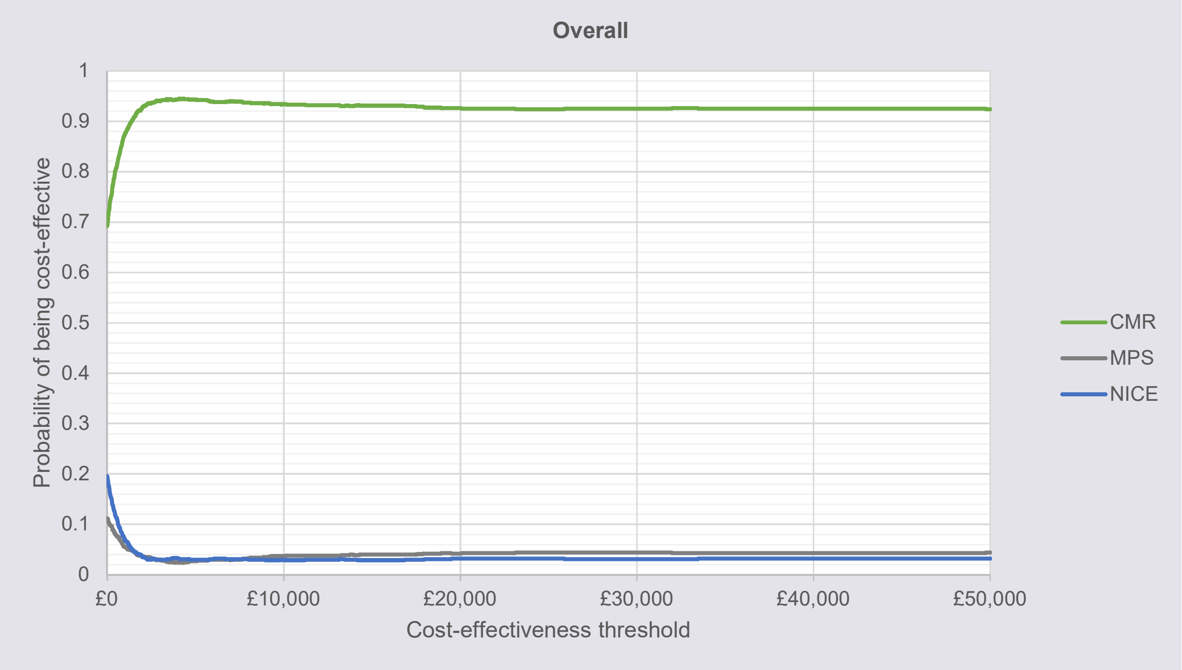

PTL: Pre-test likelihood; MPS: Myocardial Perfusion Scintigraphy; CMR: Cardiovascular Magnetic Resonance; NICE: National Institute for Health and Social Care Excellence Guidance
